# TRIM21-mediated PRMT1 degradation attenuates colorectal cancer malignant progression

**DOI:** 10.1038/s41419-025-07383-9

**Published:** 2025-01-31

**Authors:** Menghan Cao, Zhiying Shao, Xingyou Qian, Miaolei Chen, Chuyin Deng, Xintian Chen, Tingting Tang, Kaixu Zhang, Sufang Chu, Junnian Zheng, Jin Bai, Zhongwei Li

**Affiliations:** 1 Nanjing Medical University, Nanjing, Jiangsu China; 2 Cancer Institute, Xuzhou Medical University, Xuzhou, Jiangsu China; 3 Center of Clinical Oncology, the Affiliated Hospital of Xuzhou Medical University, Xuzhou, Jiangsu China; 4 Department of Clinical Trial, Zhejiang Cancer Hospital, Hangzhou Institute of Medicine (HIM), Chinese Academy of Sciences, Hangzhou, Zhejiang China; 5 Department of Hematology, Lishui City People’s Hospital, Lishui, Zhejiang China; 6 Department of Gastroenterology, the Affiliated Hospital of Guangdong Medical University, Zhanjiang, Guangdong China; 7 Laboratory of Epigenetic Regulation in Molecular Medicine, School of Basic Medical Sciences, Wannan Medical College, Wuhu, Anhui China; 8 Department of Pathophysiology, School of Basic Medical Sciences, Wannan Medical College, Wuhu, Anhui China; 9 Anhui Province Key Laboratory of Basic Research and Transformation of Age-related Diseases, Wannan Medical College, Wuhu, Anhui China

**Keywords:** Colon cancer, Tumour-suppressor proteins, Ubiquitylation, Ubiquitin ligases, Metastasis

## Abstract

Tripartite motif-containing 21 (TRIM21) plays a crucial role in antiviral responses and autoimmune diseases. While the impact of TRIM21 on cancer has been studied in various tumors, its role in colorectal cancer (CRC) remains unclear. In this study, we found that TRIM21 expression is reduced in primary CRC tissues. Low levels of TRIM21 in CRC are associated with unfavorable clinicopathological characteristics and shorter survival. Furthermore, we demonstrate that TRIM21 suppresses the proliferation, tumorigenesis, migration, and metastasis of CRC cells by promoting the ubiquitination-mediated degradation of PRMT1. These findings suggest that TRIM21 holds potential as a valuable predictive biomarker for assessing the prognosis of CRC patients.

## Introduction

Colorectal cancer (CRC), ranking third in the global incidence and mortality of malignant tumors, accounts for approximately one-tenth of all new cancer diagnoses and cancer-related deaths worldwide each year [1]. The pathogenesis of CRC has not been fully elucidated and remains a major obstacle to CRC diagnosis and treatment. Therefore, further investigation of the mechanisms regulating CRC tumorigenesis and development will contribute to more effective and targeted diagnosis, prognosis, and treatment.

The tripartite motif-containing 21 (TRIM21, also known as Ro52) belongs to the TRIM family, which includes over 80 proteins. The TRIM21 gene is found on chromosome 11 and encodes a ubiquitously expressed protein referred to as RING-dependent E3 ligase, detectable in both the cell cytoplasm and nucleus [2]. TRIM21 protein is recognized for its involvement in inflammatory processes, cancer development, and autoimmune diseases [3–6]. In cancer development, its function can vary based on the specific effectors involved in the development of cancer and the type of cancer itself. Some studies have revealed that TRIM21 exerts tumor-suppressive effects in certain cancers (for instance, breast cancer, renal cell carcinoma, and cervical cancer) [7–9], while in other types of cancer (such as glioma, nasopharyngeal carcinoma, and liver cancer), it was reported to promote tumor growth [10–12]. Interestingly, TRIM21 is associated with a reduction in stemness and metastatic potential of colorectal cancer (CRC) cells [13–15], but also contributes to chemotherapy resistance and tumorigenesis of CRC [16, 17]. Consequently, the precise role and underlying mechanism of TRIM21 in CRC remain to be fully elucidated.

Arginine methylation is a widely observed post-translational modification (PTM) in eukaryotes that controls various biological processes [18]. This modification is catalyzed by a group of enzymes called protein arginine methyltransferases (PRMTs). Among these enzymes, protein arginine methyltransferase 1 (PRMT1) is the major type I PRMT responsible for 85% of the activity attributed to type I PRMTs in mammals. PRMT1 plays crucial roles in cellular processes such as transcriptional regulation, signal transduction, and DNA damage repair, due to its ability to modify both histone and non-histone substrates [19]. The formation of asymmetric dimethylarginines (ADMAs) by PRMT1 is involved in tumorigenesis and metastasis, making it a potential target for novel drugs [20–23]. However, despite promising results in preclinical studies, specific inhibitors of PRMT1 have not shown sufficient efficacy in clinical trials [24]. Additionally, limited information is available regarding the regulation of PRMT1, particularly its protein stability. Therefore, a comprehensive understanding of the underlying mechanisms of PRMTs is necessary for successful clinical applications.

In this study, we explore the function and underlying mechanism of TRIM21 in CRC development. For the first time, we uncovered that TRIM21, a tumor suppressor gene in colorectal cancer, binds to PRMT1 using its SPRY domain and acts as an E3 ubiquitin ligase, promoting the ubiquitination and degradation of the oncogene PRMT1 in a K48-linked manner. This mechanism plays a crucial role in inhibiting the proliferation and metastasis of colorectal cancer cells both in vitro and in vivo. The focus of this topic is to explore the clinical significance of the TRIM21-PRMT1 axis in colorectal cancer and provide new insights for the treatment of this disease.

## Materials and Methods

### Patients and sample collection

In this study, tissue samples were collected from 485 pairs of colorectal cancer (CRC) tissues at the Affiliated Hospital of Xuzhou Medical University in China, between 2005 and 2008. The clinicopathologic information of the patients was obtained from the hospital’s medical records. The researchers calculated the overall survival (OS) time based on the date of surgery to the date of death, and the disease-specific survival (DSS) time from the time of surgery until death caused by CRC.

### Cell culture and cell treatment

The HEK293T and CRC cell lines were obtained from the cell bank of the Chinese Academy of Sciences. These cells were cultured in DMEM (KGL1207-500, KeyGEN BioTECH) supplemented with 10% fetal bovine serum, 100 U/ml penicillin, and 100 μg/ml streptomycin. The cells were then incubated in a humidified incubator at 37 °C with 5% CO_2_.

In order to knock down the expression of TRIM21 in CRC cells, small interfering RNAs (siRNAs) targeting TRIM21 were transfected into the cells using siLenFect reagent. Non-specific siRNA was used as a negative control. The siRNAs used in this study were purchased from GenePharma Technology in Shanghai, China. For plasmid transfections, the researchers used Lipofectamine 2000 according to the manufacturer’s instructions.

### Western blot analysis and antibodies

Western blot analysis was performed as previously described [25]. The specific primary antibodies against TRIM21 (11039-1-AP, Proteintech), PRMT1 (11279-1-AP, Proteintech), GAPDH (60004-1-AP, Proteintech), Cyclin D1 (2978 T, Cell Signaling Technology), Cyclin E2 (4132 T, Cell Signaling Technology), p21 (10883-1-AP, Proteintech), p16 (10355-1-AP, Proteintech), E-cadherin (610181, BD Biosciences, Bedford, Massachusetts, USA), N-cadherin (610920, BD Biosciences, Bedford, Massachusetts, USA), Snail (A11794, Abclonal), flag (20543-1-AP, Proteintech), HA (51064-2-AP, Proteintech), and ubiquitin (58395S, Cell Signaling Technology), His (66005-1-Ig, Proteintech), myc (60003-2-Ig, Proteintech), were used for western blot assays.

### RNA extract, reverse transcription-PCR and qRT-PCR

The extraction of RNA was performed by utilizing TRIzol (Invitrogen), and the synthesis of cDNA was conducted with the HiScript 1st Strand cDNA Synthesis Kit (Vazyme Biotech, Nanjing, China). Realtime PCR was executed on the ABI-7500 instrument employing the UltraSYBR One Step RT-qPCR Kit (CWBIO, Beijing, China). The primers utilized for quantitative RT-PCR analysis can be found in the Supplementary materials.

### Immunohistochemistry (IHC)

IHC assays were conducted using the standard streptavidin-peroxidase method as previously described [26]. The primary antibody utilized was the anti-TRIM21 antibody, diluted at a ratio of 1:400. For the anti-PRMT1 antibody, a dilution of 1:100 was employed. As for the anti-Ki-67 antibody (12202S, CST), a dilution of 1:100 was utilized. To serve as a negative control, a slide without primary antibody incubation was included.

### Cell proliferation migration, invasion, and wound healing assays

The cell proliferation was assessed using the Cell Counting Kit-8 (CCK-8) assay based on the protocol provided by the manufacturer (Dojindo). Cell migration and invasion assay was performed as previously described [27].

For the wound healing assay, cells were seeded in six-well plates and allowed to reach approximately 80% confluence. Subsequently, an artificial scratch was created in each well using a sterile 10-μl pipette tip. The PBS solution was used to wash away any detached cells. Following that, the cells were cultured in a medium containing 1% FBS. The migration distance of the cells was documented under an inverted light microscope at both the 0 and 24-hour time points.

### Stable cell line generation

To generate four distinct HCT-116 stably transfected cell lines (OE-TRIM21 or OE-PRMT1), we employed lentiviral packaging and transfection techniques. The methodology for producing stable cells using lentivirus was demonstrated as previously reported [21].

### Animal work

BALB/c nude mice (6–8 weeks of age) were obtained from Beijing Vital River Laboratory Animal Technology Co., Ltd. (Beijing, China). Our research involving animals received approval from the Animal Care and Use Committee at Xuzhou Medical University. Four distinct HCT-116 stably transfected cell groups consisted of: a control group (NC) with vector+vector, an overexpression group of TRIM21 (OE-TRIM21) with vector+OE-TRIM21, an overexpression group of PRMT1 (OE-PRMT1) with vector+OE-PRMT1, and a co-overexpression group of TRIM21 and PRMT1 (OE-TRIM21 + OE-PRMT1). For the subcutaneous tumor model, 5 × 10^6^ cells were respectively inoculated into the ventral region of the mice. Tumor volume was calculated on a weekly basis using the formula V = a × (b × b)/2, where ‘a’ represents the largest diameter and ‘b’ represents the smallest diameter. In the lung metastasis model, 2 × 10^6^ cells were injected into the mice via the caudal vein. After ten weeks of injection, the nude mice were euthanized, and their lung tissues were dissected and removed for bouin staining.

### Statistical analysis

Statistical analyses were conducted using SPSS 20.0 software (SPSS Inc., Chicago, IL, USA) and GraphPad Prism 7. The relationship between TRIM21 and the clinicopathologic parameters of CRC patients was assessed utilizing a Chi-square test. To assess the correlation between TRIM21 expression and CRC patient survival, the Kaplan-Meier method and log-rank test were employed. Statistical analysis involving three sets of single factor analysis was conducted through one-way ANOVA, which was corrected using Tukey’s multiple comparison test for single factor comparison. Mean ± standard deviation was used to present the data, with the bar representing the mean value. To ascertain statistical differences between the control and overexpression groups, a two-tailed Student’s t-test was employed. Mean squared error (SEM) was presented as data, with the bar indicating the mean. A p-value of less than 0.05 was considered statistically significant.

## Results

### Low TRIM21 expression correlates with clinicopathologic parameters and poor survival in CRC patients

**Fig. 1:**
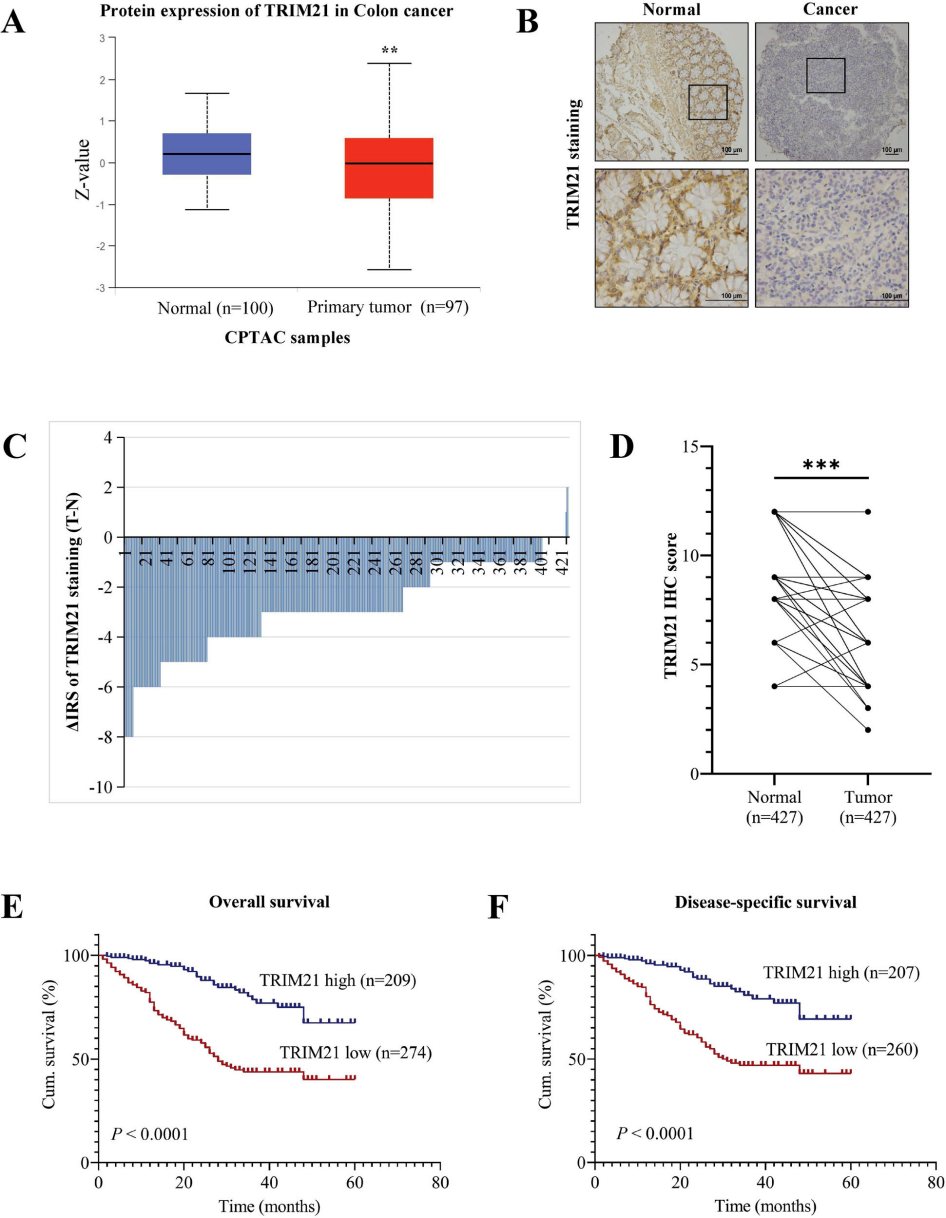
TRIM21 expression is decreased and correlates with poor survival in CRC patients. **A** Protein expression level of TRIM21 in normal colon tissues and colon cancer tissues. Data were obtained from CPTAC database. **B** Representative immunohistochemistry images of TRIM21 protein expression in 427 paired adjacent non-cancerous tissue and CRC tissues. **C**, **D** Staining intensities (**C**) and immunohistochemistry score (**D**) of TRIM12 in CRC tissues compared with paired adjacent non-cancerous tissue. (N, paired adjacent non-cancerous tissues. T, tumor tissues). **E**, **F** The Kaplan–Meier survival curves of overall survival (**E**) and disease-specific survival (**F**) in patients with CRC stratified by Trim21 protein expression levels in CRC tissues. Data were expressed as the mean ± SD (**P < 0.01, *** *P* < 0.001).

To investigate the role of TRIM21 in CRC, we utilized the Clinical Proteomic Tumor Analysis Consortium (CPTAC) database to analyze the protein expression level of TRIM21 in colon cancer patients. The findings revealed a significant decrease in TRIM21 expression in colon cancer tissues compared to normal tissues (Fig. 1A). To confirm these findings, we performed IHC staining of TRIM21 protein on tissue chips containing 427 CRC tissue samples and their paired adjacent tissues. The staining results consistently supported the database analysis, demonstrating lower TRIM21 expression in colorectal cancer compared to normal tissue (Fig. 1B–D). Additionally, we employed Fisher’s exact test to examine the correlation between TRIM21 expression in CRC and clinicopathological characteristics (Table 1) to gain further insights into the clinical significance of TRIM21 in CRC. The results indicated a significant positive correlation between low TRIM21 expression and large tumor size (P < 0.001), poor tumor differentiation (P < 0.001), lymph node metastasis (P < 0.001), distant metastasis (P < 0.001), and advanced TNM stage (P < 0.001). However, no association was found between TRIM21 expression and age or gender.

**Table 1:** Relationship between TRIM21 expression and clinicopathological features of CRC patients.

Variables	TRIM21 expression (*n* = 485)		
	Low (%)	High (%)	P*
**Age (years)**			0.455
≤56	86 (54.1)	73 (45.9)	
>56	188 (57.7)	138 (42.3)	
**Gender**			0.284
Males	149 (54.4)	125 (45.6)	
Females	125 (59.2)	86 (40.8)	
**Tumor diameter**			< 0.001
<5 cm	176 (49.7)	178 (50.3)	
≥5 cm	98 (75.4)	32 (24.6)	
**Differentiation**			< 0.001
Poor	72 (75.0)	24 (25.0)	
Moderate/high	198 (51.6)	186 (48.4)	
**Lymph node metastasis**			< 0.001
Negative	126 (45.7)	150 (54.3)	
Positive	135 (68.9)	61 (31.1)	
**Distant metastasis**			< 0.001
M0	253 (54.5)	211 (45.5)	
M1	21 (100.0)	0 (0.0)	
**TNM stage**			< 0.001
I / II	119 (44.2)	150 (55.8)	
III / IV	155 (71.8)	61 (28.2)	

^*^P values are from χ^2^ test.

The Kaplan–Meier survival analysis and log-rank test were used to investigate the correlation between TRIM21 expression and OS and DSS in CRC patients. The data revealed that patients with low TRIM21 expression had worse OS and DSS compared to those with high TRIM21 expression (Fig. 1E and F). To further assess the independent prognostic value of TRIM21 expression in CRC, univariate and multivariate Cox regression models were employed. The results of the univariate Cox regression analysis indicated that TRIM21 expression, tumor differentiation, lymph node metastasis, distant metastasis, and TNM stage were significant prognostic factors for both OS and DSS in CRC patients (Table S1). The multivariate Cox regression model confirmed that TRIM21 expression was an independent prognostic biomarker for OS and DSS in CRC patients (Table S2). In conclusion, our findings demonstrate that TRIM21 is expressed at lower levels in CRC tissues and may serve as a potential independent prognostic factor for CRC patients.

### TRIM21 inhibits the proliferation and motility of CRC cells in vitro

**Fig. 2:**
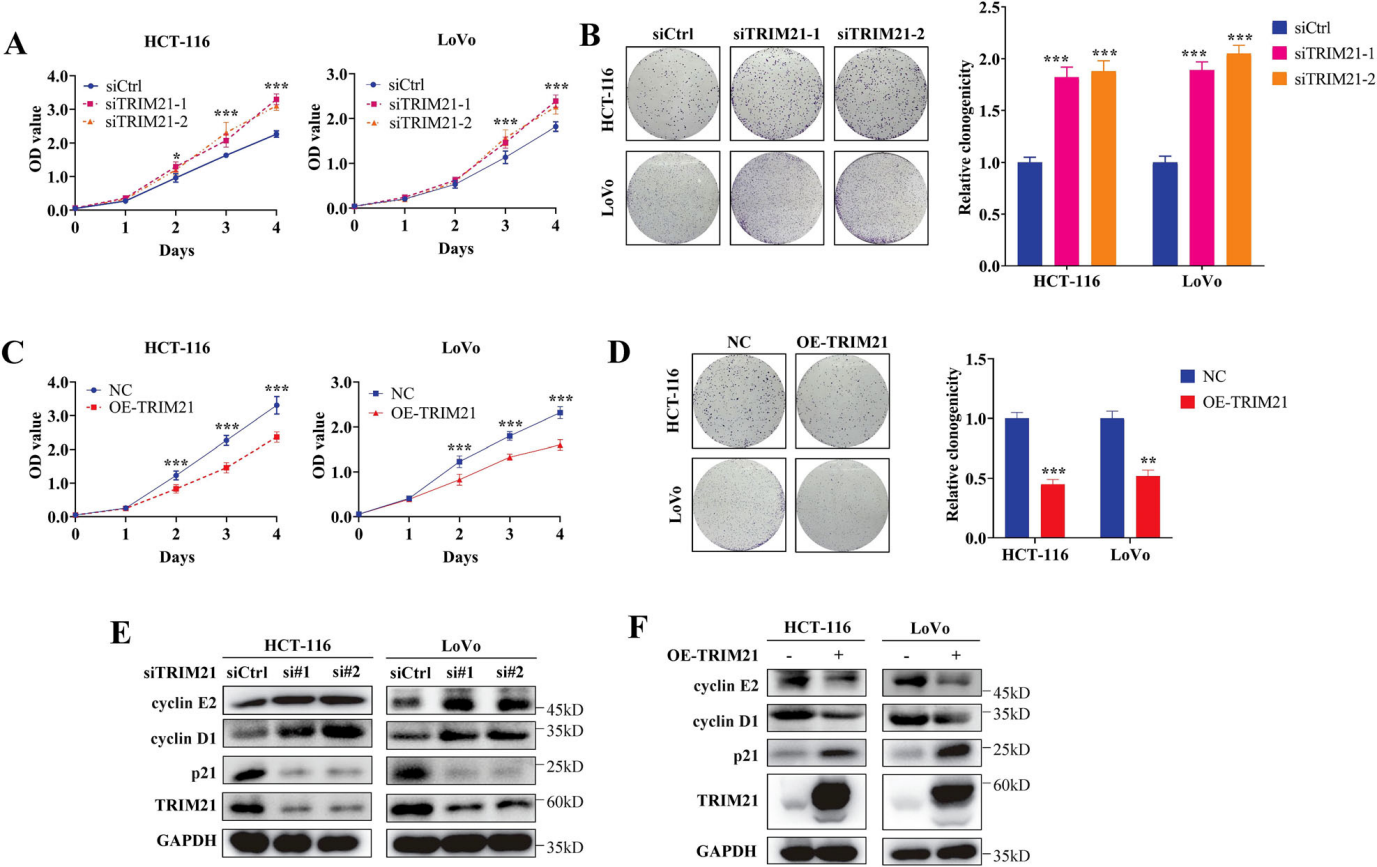
TRIM21 inhibits the proliferation of CRC cells in vitro. **A–D** CCK-8 and colony formation assays were used to assess the effect of TRIM21 knockdown **(A**, **B)** or overexpression **(C, D)** on cell proliferation in HCT-116 and LoVo cell lines. **E, F** The impact of TRIM21 knockdown (**E**) or overexpression (**F**) on the major cyclins were detected by western blot analysis. Data are represented as mean ± SD of three independent experiments, and ***p < 0.001 (Student’s t-test).

To further investigate the function of TRIM21 in CRC cells, we conducted knockdown and overexpression experiments in HCT-116 and LoVo cells. The results from CCK-8 and clone formation analysis showed that the proliferation rate was accelerated when TRIM21 was knocked down (Fig. 2A and B), but decreased when TRIM21 was overexpressed (Fig. 2C and D).

P21 is the first identified cyclin-dependent kinase regulator that inhibits G1/S cell cycle progression. Reports indicate that TRIM21 may either increase or decrease the expression levels of P21 across various tumors [28, 29]. Consequently, the impact of TRIM21 on the cell cycle remains a contentious issue. Here, we hypothesized that TRIM21 may suppress tumor proliferation by influencing the cell cycle process. We conducted a western blot analysis to investigate the expression levels of p21 and two major cyclins, cyclin D1 and cyclin E2. Cyclin D1 acts as an essential positive regulator of the cell cycle, promoting accelerated and uncontrolled proliferation by shortening the G1 phase and causing early entry into the S phase. Cyclin E2, a major rate-limiting factor in the G1/S transition, is overexpressed in various tumors and functions by promoting cancer. The results of our analysis indicated that TRIM21 deficiency led to increased levels of cyclin E2 and cyclin D1, but decreased levels of p21 (Fig. 2E). Conversely, excessive expression of TRIM21 showed the opposite results (Fig. 2F).

**Fig. 3:**
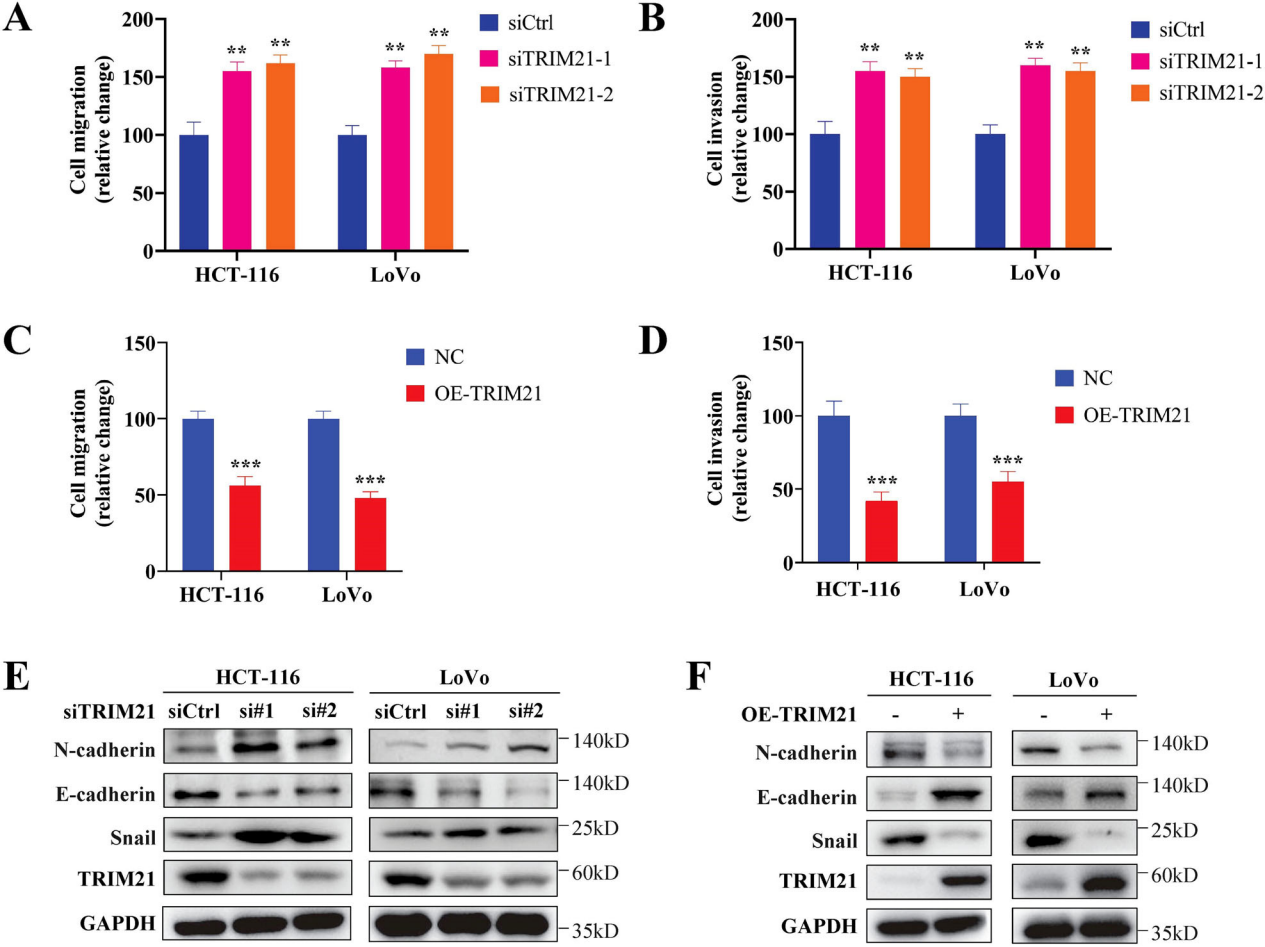
TRIM21 restricts the motility of CRC cells in vitro*.* **A** Transwell migration assays and Matrigel invasion assays were used to detect the migration and invasion ability of CRC cells with TRIM21 deficiency (**A**, **B**) or TRIM21 overexpression (**C**, **D**). **E, F** Western blot analysis of EMT markers in TRIM21 knockdown (**E**) or TRIM21 overexpression CRC cells (**F**). Data are represented as mean ± SD of three independent experiments, and **p < 0.01, ***p < 0.001 (Student’s t-test).

In terms of tumor motility, transwell analysis demonstrated that TRIM21 knockdown promoted the migration and invasion ability of CRC cells (Fig. 3A and B), while TRIM21 overexpression inhibited these abilities (Fig. 3C and D). Correspondingly, TRIM21 deficiency led to an elevated level of N-cadherin and Snail, but a diminished level of E-cadherin (Fig. 3E). The results were converse with TRIM21 overexpression (Fig. 3F). Overall, our findings suggest that TRIM21 inhibits the proliferation and motility of CRC cells in vitro.

### TRIM21 binds to PRMT1 and reduces its protein stability

To investigate the tumor suppressor effect of TRIM21 in colorectal cancer, we transfected control vector plasmid and TRIM21 overexpression plasmid in HCT-116 cells. The resulting protein samples were then analyzed using mass spectrometry to identify potential downstream targets of TRIM21. Among the 760 candidate targets, our attention was drawn to the arginine methyltransferase, PRMT1. Previous studies have highlighted the high expression and cancer-promoting role of PRMT1 in cancer [30–32]. Furthermore, our research group has previously discovered that PRMT1 mediates asymmetric methylation modification on the R342 site of EZH2, leading to enhanced stability of the EZH2 protein and promotion of breast cancer metastasis [21]. Additionally, PRMT1 can promote the proliferation of breast cancer cells by inhibiting the transcription and expression of p16 and p21 [33].

**Fig. 4:**
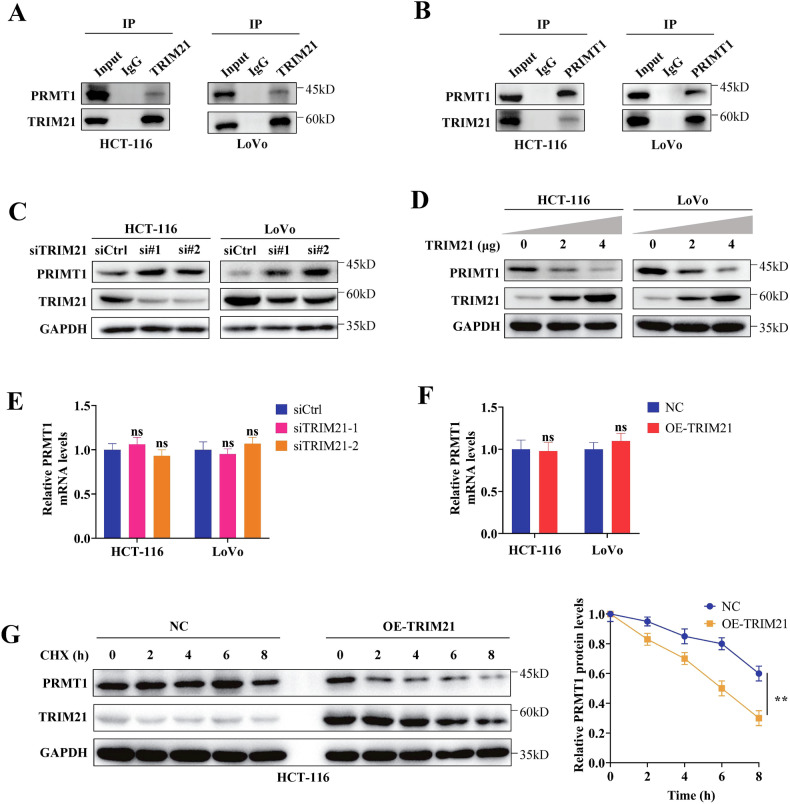
TRIM21 decreases the protein stability of PRMT1 in CRC cells. **A, B** The mutual interaction between TRIM21 and PRMT1 were detected by Co-IP. **C, D** Western blot analysis was used to detect PRMT1 protein expression in TRIM21 deficiency (**C**) or TRIM21 overexpression (**D**) cells. **E, F** qPCR analysis was used to detect PRMT1 mRNA expression in TRIM21 deficiency (**E**) or TRIM21 overexpression (**F**) cells. **G** Effect of TRIM21 on PRMT1 protein half-life in HCT-116 cells overexpressing TRIM21 and treated with CHX. Data are represented as mean ± SD of three independent experiments, and ns (no significance), **p < 0.01 (Student’s t-test).

To investigate the potential interaction between TRIM21 and PRMT1, we initially conducted Co-IP experiments to confirm the mutual binding of endogenous TRIM21 and PRMT1 in HCT-116 and LoVo cells (Fig. 4A and B). Subsequently, western blot experiments were performed, revealing that TRIM21 negatively regulates the protein level of PRMT1 in HCT-116 and LoVo cells (Fig. 4C and D). However, the mRNA levels of PRMT1 were not significantly affected by TRIM21 knockdown or overexpression (Fig. 4E and F), indicating that TRIM21 primarily regulates the post-translational modification of PRMT1. Treatment of HCT-116 cells with the protein synthesis inhibitor cycloheximide (CHX) demonstrated that the degradation rate of the PRMT1 protein was higher in the TRIM21 overexpression group compared to the control group, resulting in a shortened half-life (Fig. 4G). These findings suggest that TRIM21 reduces the stability of PRMT1 in CRC cells.

### TRIM21-mediated K48-linked ubiquitination degradation of PRMT1 in CRC cells

**Fig. 5:**
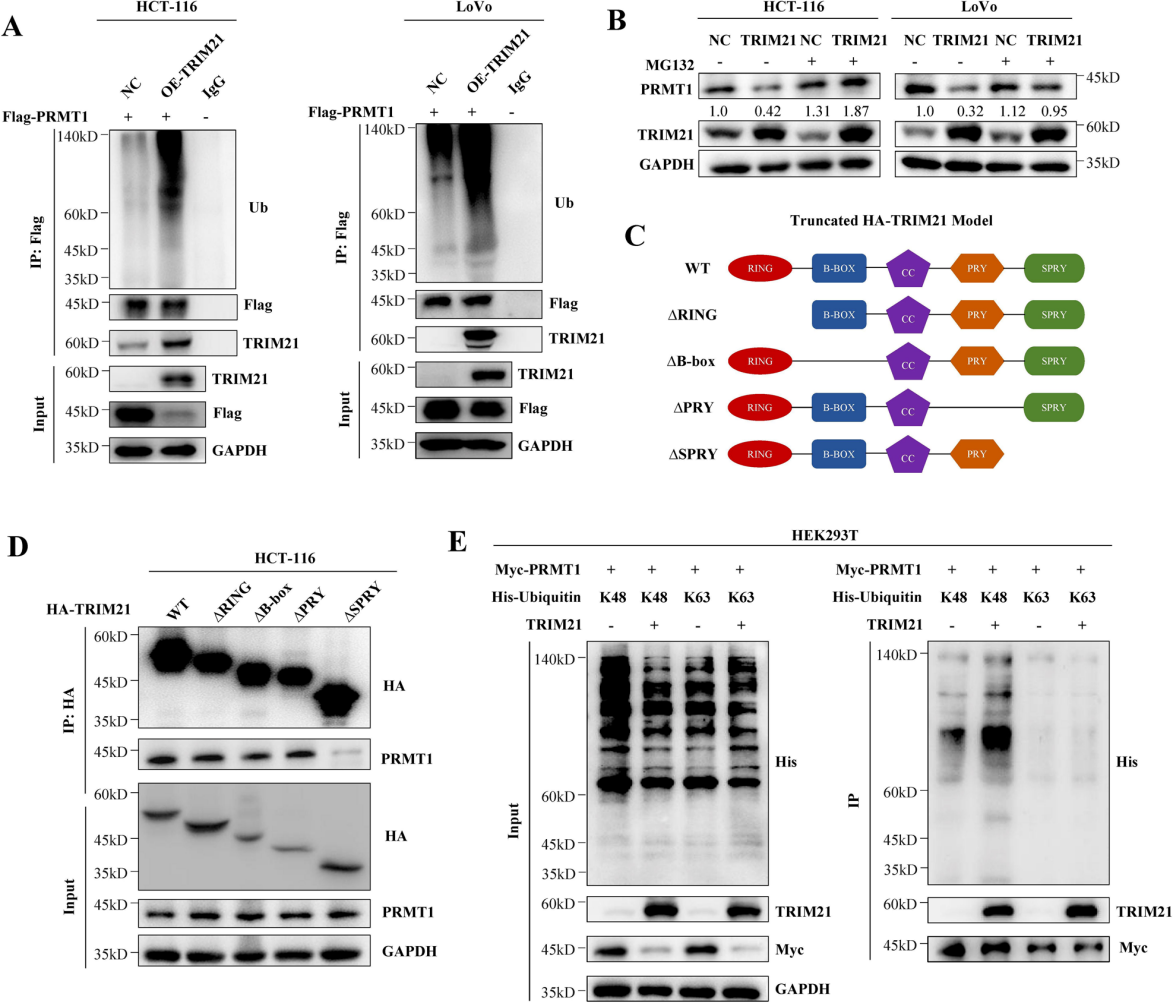
TRIM21mediates K48-linked ubiquitination degradation of PRMT1 in CRC cells. **A** TRIM21 mediated ubiquitination degradation of PRMT1 in HCT-116 and LoVo cells. **B** Western blot analysis showing the effect of the proteasome inhibitor MG132 (10 μM for 6 h) treatment on PRMT1 protein accumulation in HCT-116 and LoVo cells. The relative intensity of PRMT1 proteins were quantified by software Image J. **C** Schematic representation of TRIM21 truncated mutations. **D** HCT-116 cells were transfected with wild-type TRIM21 or the indicated mutants to detected the potential domain that interacted with PRMT1. **E** K48-only and K63-only ubiquitin plasmids alone or co-transfected with TRIM21-HA plasmid into 293 T cells for 48 h, and the cells were then harvested and subjected to myc IP.

TRIM21, an E3 ubiquitin ligase, is known to be involved in various crucial biological functions by facilitating ubiquitination modification. To investigate whether TRIM21 mediates the ubiquitination and degradation of PRMT1 through the ubiquitination-proteasome pathway, we introduced the Flag-PRMT1 plasmid into HCT-116 and LoVo cells and subsequently transfected the control plasmid and TRIM21 overexpression plasmid. Co-IP analysis revealed that the ubiquitination level of PRMT1 was significantly higher in the TRIM21 overexpression group compared to the control group, indicating that TRIM21 can mediate the ubiquitination modification of PRMT1 in CRC cells (Fig. 5A). After adding MG132, a proteasome inhibitor, the down-regulating effect of TRIM21 on PRMT1 protein levels was reversed (Fig. 5B and Figure S1A), suggesting that TRIM21 regulates PRMT1 protein stability through the ubiquitination-proteasome pathway.

The structure of TRIM21 contains the C-terminal RING-finger domain, the zinc finger domain (B-box) and the coiled coil (CC) region, and the N-terminal contains the SPRY domain and the PRY domain [34]. To determine the domain responsible for PRMT1 interaction and degradation, we utilized several domain-truncated HA tag TRIM21 (Truncated HA-TRIM21) (Fig. 5C) [34, 35]. Our findings revealed that truncated mutants lacking the SPRY domains exhibited impaired ability to interact with PRMT1 (Fig. 5D), indicating that SPRY is primarily required for the interaction.

There are various types of ubiquitination modifications, categorized based on the different lysine residue positions of the connected ubiquitin molecules. Among these, polyubiquitination at K48 and K63 sites is the most prevalent. Previous studies have indicated that K48-linked polyubiquitination primarily regulates protein degradation and stability, whereas K63-linked polyubiquitination primarily regulates intracellular signal transduction [36]. Co-IP assays confirmed that TRIM21 predominantly induced K48-linked ubiquitination of PRMT1 (Fig. 5E).

Collectively, these results demonstrate that TRIM21 has a universal regulatory effect on PRMT1 ubiquitination in CRC cells. The interaction between TRIM21’s SPRY domain and PRMT1 facilitates the delivery of K48-ubiquitin to PRMT1, resulting in the recognition and degradation of PRMT1 by proteasomes.

### PRMT1 is required for TRIM21-mediated CRC progression in vitro

**Fig. 6:**
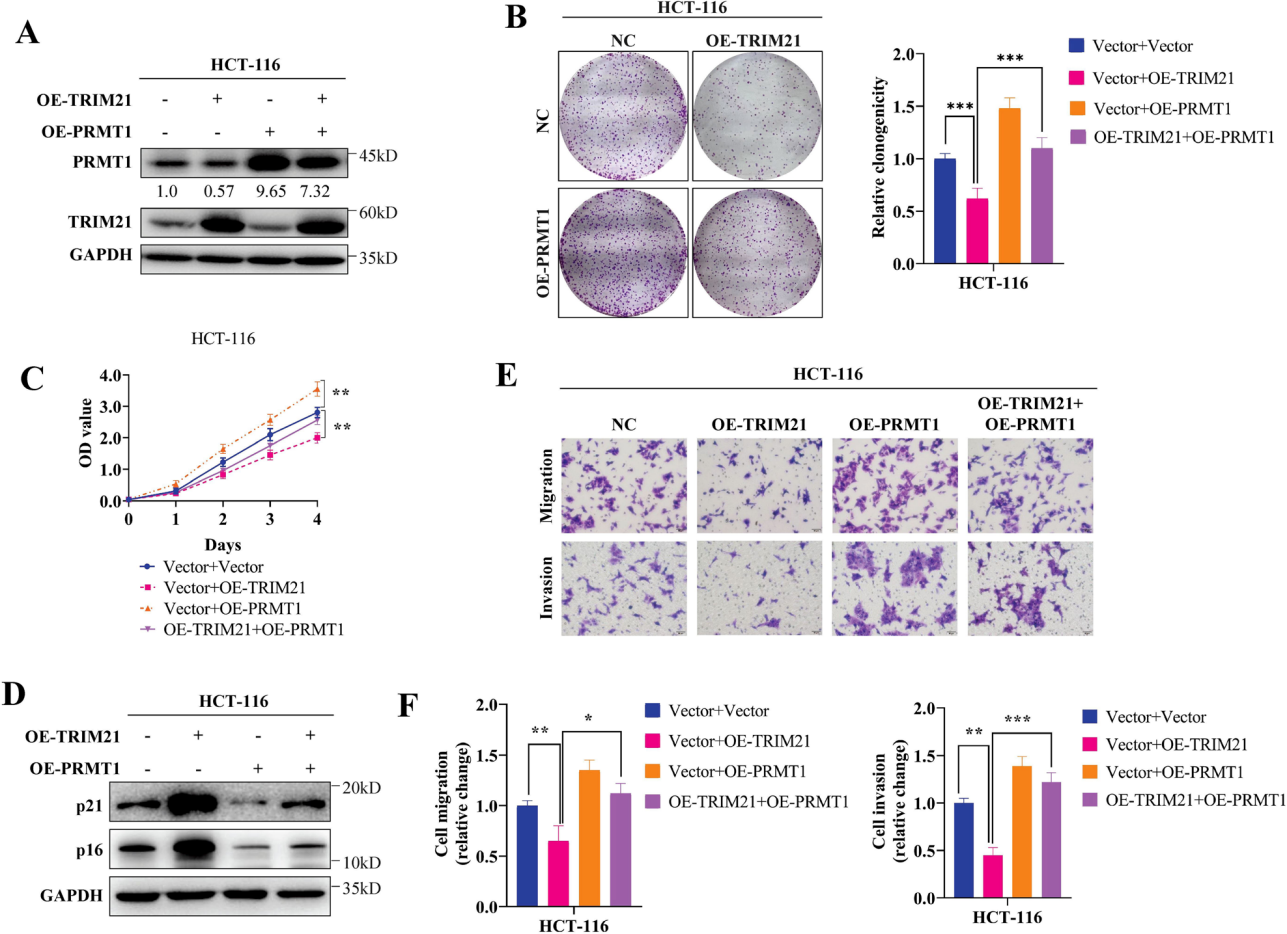
PRMT1 is required for TRIM21-mediated CRC progression in vitro*.* **A** Western blot was used to detect the overexpression efficiency of TRIM21 and PRMT1 in HCT-116 cells. The relative intensity of PRMT1 proteins were quantified by software Image J. **B, C** Clone formation assays (**B**) and CCK-8 assays (**C**) were used to assess the effect of TRIM21 overexpression, PRMT1 overexpression or both on HCT-116 cell proliferation. **D** Detection of p16 and p21 expression in TRIM21 overexpression HCT116 cells after over-expressing PRMT1 or not. **E, F** Representative images (**E**) and statistical results (**F**) of transwell migration assays and Matrigel invasion assays to assess the migration and invasion ability of HCT-116 cells with TRIM21 overexpression, PRMT1 overexpression or both.Data are represented as mean ± SD of three independent experiments, and *p < 0.05, **p < 0.01, ***p < 0.001 (Student’s t-test).

Previous studies conducted by our research team have revealed that PRMT1 plays a role in promoting the proliferation and metastasis of both breast cancer and colorectal cancer. In the present study, we discovered that TRIM21 has the ability to inhibit the proliferation and metastasis of CRC. Meanwhile, TRIM21 can ubiquitinate and degrade PRMT1. Based on these findings, we hypothesize that TRIM21 may act as a tumor suppressor in CRC by negatively regulating PRMT1. The transfection efficiency of TRIM21 and PRMT1 in the four groups of HCT-116 cells was confirmed through western blot experiments, demonstrating the successful construction of the cell lines (Fig. 6A and Figure S1B).

The results of CCK-8 and colony formation assays indicated that PRMT1 overexpression promoted cell proliferation. Conversely, TRIM21 overexpression inhibited cell proliferation, which could be reversed by overexpression of PRMT1 in cells that also overexpressed TRIM21 (Fig. 6B and C). Furthermore, our western blot data showed that p16 and p21 were strongly increased after over-expressing TRIM21 in HCT-116 cells, but ectopic expression of PRMT1 weakened the increased amount of p16 and p21 caused by TRIM21 overexpression (Fig. 6D). Subsequently, transwell experiments were conducted on four groups of cells to evaluate their migration and invasion abilities. The results revealed that TRIM21 overexpression hindered cell migration and invasion, while PRMT1 overexpression facilitated these processes. Notably, in cells overexpressing TRIM21, the migration inhibitory effect was restored upon PRMT1 overexpression (Fig. 6E and F). These findings suggest a PRMT1-dependent mechanism by which TRIM21 inhibits the proliferation and metastasis of CRC cells in vitro.

### TRIM21 affects CRC development in a PRMT1-dependent manner in vivo

**Fig. 7:**
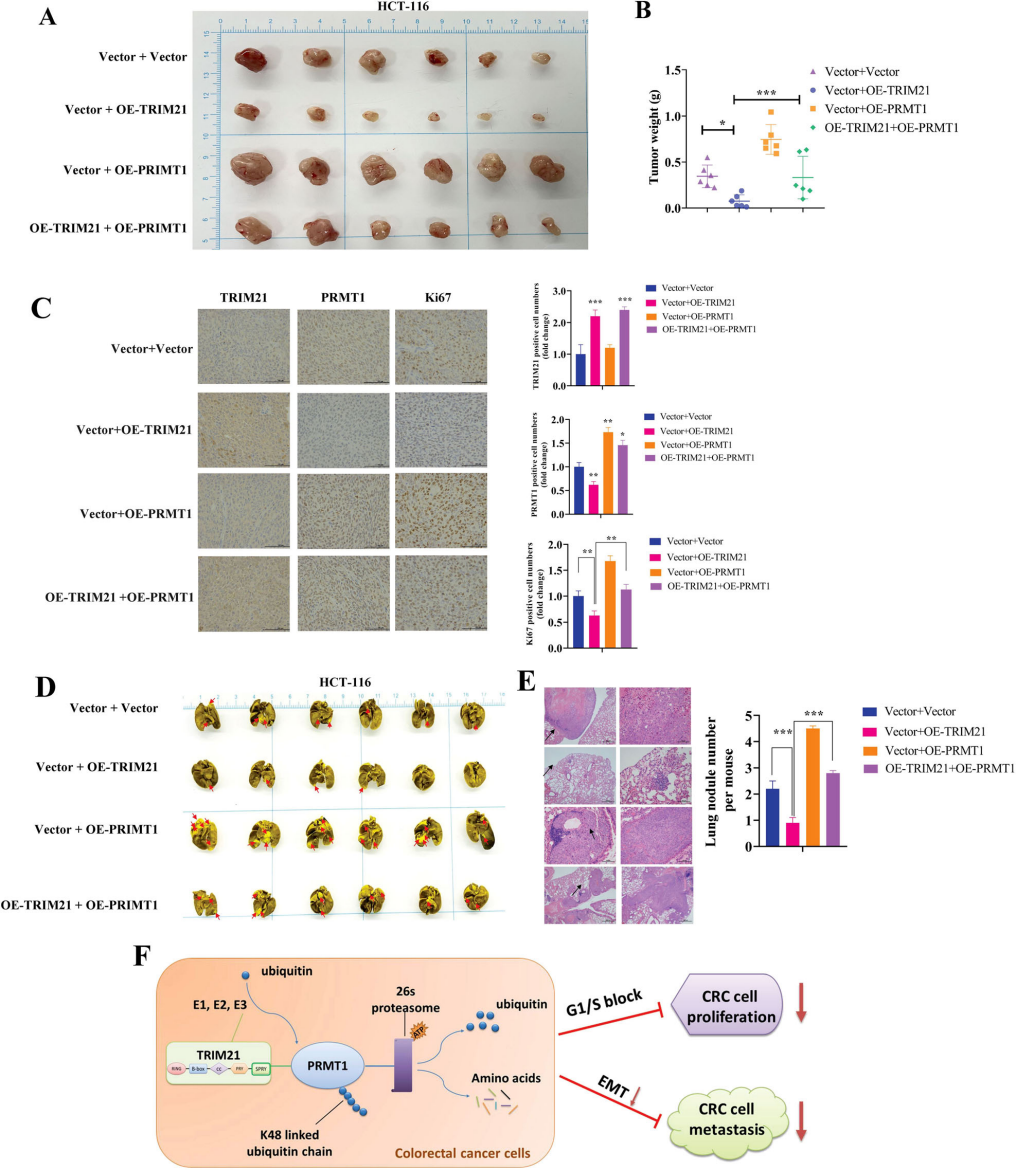
TRIM21 affects the CRC progression in a PRMT11-dependent manner in vivo*.* **A, B** Tumor tissue images (**A**), tumor volume statistical curves (**B**) of HCT116 tumor-bearing mice in indicated groups (*n* = 6 for each group). **C** IHC staining was performed on the subcutaneous tumor tissue to analyze the expression of TRIM21, PRMT1, and Ki-67. **D** Lung metastatic nodules were examined macroscopically after nude mice were sacrificed. The red arrows denote the metastatic nodules. **E** Bouin staining and HE staining of lung metastases after tail vein injection of BALB/C nude mice (Left panel). statistical analysis of lung nodule numbers (Right panel). Data were expressed as the mean ± SD (*P < 0.05, **P < 0.01, *** *P* < 0.001). **F** A proposed working model for this study. TRIM21-mediated PRMT1 K48-linked ubiquitination decreases PRMT1 expression, which suppresses the proliferation, tumorigenesis, migration, and metastasis of CRC cells.

To investigate the potential inhibitory effects of TRIM21 on CRC tumorigenesis and metastasis in vivo, we conducted two models: a nude mouse subcutaneous tumor model and a tail vein lung metastasis model. In the subcutaneous tumor model, we observed that the tumor weight and volumes were significantly lower in the OE-TREIM21 group compared to the control group. However, in the OE-PRMT1 group, the tumor weight and volumes were higher. To determine if TRIM21 functions in a PRMT1-dependent manner, we restored the expression of PRMT1 in the OE-TRIM21 group, which reversed the tumor-inhibiting effect of TRIM21 (Fig. 7A and B). IHC staining was also performed on the subcutaneous tumor tissue to analyze the expression of TRIM21, PRMT1, and Ki-67 (Fig. 7C). The results showed stable overexpression of TRIM21 and PRMT1 in the corresponding groups. Ki67 staining, an important indicator of cell proliferation, revealed a significantly lower number of Ki67-positive cells in the OE-TRIM21 group compared to the control group. Conversely, the OE-PRMT1 group had a higher number of Ki67-positive cells. Interestingly, the OE-TRIM21 + OE-PRMT1 group showed a significant increase in the number of Ki67-positive cells compared to the OE-TRIM21 group.

For the lung metastasis model, bouin staining revealed that the number of intrapulmonary metastases was significantly reduced in the OE-TRIM21 group compared to the control group, while it increased in the OE-PRMT1 group. Moreover, the number of metastases was significantly higher in the OE-TRIM21 + OE-PRMT1 group compared to the OE-TRIM21 group (Fig. 7D and E). The above findings suggest that TRIM21 can inhibit the tumorigenic and metastatic ability of CRC cells in vivo in a PRMT1-dependent manner.

## Discussion

The prevalence of colorectal cancer has been increasing in recent years, making it currently the third most common malignant tumor [37]. Despite the development and clinical application of new drugs such as targeted therapies and immune checkpoint inhibitors, the cure rate for colorectal cancer has not significantly improved resulting in a high mortality rate, which is the second leading cause of cancer death overall [1]. Although some patients with left colon disease may experience symptoms of malignant intestinal obstruction in the early stages, most patients do not show obvious symptoms initially. The insidious onset of intestinal cancer leads to delayed diagnosis, often when the cancer has already advanced with deep local invasion, extensive intra-abdominal lymph node metastasis, or even distant metastasis. Therefore, it is crucial to identify sensitive and specific biomarkers for CRC and discover more effective therapeutic targets in CRC research.

The role of TRIM21 in regulating innate immunity and its involvement in autoimmune diseases, antiviral processes, and other biological processes have been extensively studied by scholars. However, recent research has also shed light on the role of TRIM21 in malignant tumors. This study discovered a reduction of TRIM21 expression in intestinal cancer tissues compared to para-cancerous tissues. Furthermore, the low expression of TRIM21 was found to be correlated with various clinicopathological parameters, and act as an independent prognostic factor in patients with CRC.

Subsequently, we conducted mass spectrometry analysis to identify potential downstream targets of TRIM21, aiming to investigate its mechanism of action. Among the candidate genes, PRMT1 drew our attention. Previous research conducted by our research team has demonstrated that PRMT1 can enhance the proliferation of breast cancer cells by inhibiting the transcription and expression of p16 and p21 [33]. Meanwhile, PRMT1 can facilitate the asymmetric methylation modification of EZH2, enhancing its protein stability and promoting the EMT process of breast cancer cells, which ultimately leads to breast cancer metastasis [21]. In our project, TRIM21 was found to positively regulate the protein levels of p21 but negatively regulate the protein levels of Cyclin E2 and Cyclin D1. Further in vitro and in vivo experiments confirmed the tumor suppressor role of TRIM21 in inhibiting CRC proliferation and metastasis. Therefore, we hypothesized that TRIM21 may function in a PRMT1-dependent manner. In term of mechanism, TRIM21 was found to interact with PRMT1 and negatively regulated the protein stability of PRMT1 by facilitating its degradation through the K48-linked ubiquitin-proteasome pathway. Notably, the SPRY domain of TRIM21 serves as the binding region for PRMT1, while the specific domain on PRMT1 that interacts with TRIM21 warrants further investigation in future studies.

To verify whether TRIM21 acts its tumor suppressor function via PRMT1, we overexpressed PRMT1 in CRC cells with a base of TRIM21 overexpression. The subsequent in vitro and in vivo experiments consistently demonstrated that the restoration of PRMT1 expression reversed the inhibitory effect of TRIM21 on the proliferation and metastasis of CRC cells. Further investigation is required to elucidate the downstream targets and pathways involved in the interaction between TRIM21 and PRMT1 in CRC.

In this study, we investigated the role of TRIM21 in mediating the degradation of PRMT1 through ubiquitination. Our findings revealed that this process resulted in a decrease in PRMT1 protein expression and subsequently inhibited the growth and metastasis of CRC cells (Fig. 7F). These results provide insights into the underlying mechanisms of PRMT1 and offer potential solutions for the challenges associated with anti-PRMT1 therapy. Additionally, our study emphasizes the possibility of developing targeted drugs that specifically activate TRIM21, presenting a promising therapeutic approach for the treatment of colorectal cancer and opening up new avenues for clinical intervention.

## Supplementary information

Supplymentary Table1

Supplymentary Table2

Supplymentary Figure 1

Supplementary Material and Methods

uncropped WB Raw Data

## Data Availability

The data supporting the findings of this study are available from the corresponding author upon reasonable request.
