# Supplementary material for: TRIM21-mediated PRMT1 degradation attenuates colorectal cancer malignant progression
Source: Cell Death Dis. 2025 Jan 31;16(1):56. doi: 10.1038/s41419-025-07383-9 (PMC11785787; doi:10.1038/s41419-025-07383-9)
Supplement: Supplementary file 1 — Supplymentary Table1 [file 41419_2025_7383_MOESM1_ESM.docx]

**Table S1** Univariate Cox proportional regression analysis of overall survival and disease-specific survival of CRC patients.

| **Variable** | **Overall survival** | | |  | **Disease-specific survival** | | |
| --- | --- | --- | --- | --- | --- | --- | --- |
|  | **Hazard ratio** | **95% CI^†^** | ***P*^*^** |  | **Hazard ratio** | **95% CI^†^** | ***P*^*^** |
| **TRIM21 staining** | |  |  |  |  |  |  |
| Low | 1.000 |  | < 0.001 |  | 1.000 |  | < 0.001 |
| High | 0.260 | 0.171-0.393 |  |  | 0.271 | 0.176-0.417 |  |
| **Age** |  |  |  |  |  |  |  |
| ≤ 56 years | 1.000 |  | 0.151 |  | 1.000 |  | 0.271 |
| > 56 years | 1.303 | 0.908-1.870 |  |  | 1.237 | 0.847-1.806 |  |
| **Gender** |  |  |  |  |  |  |  |
| Male | 1.000 |  | 0.013 |  | 1.000 |  | 0.054 |
| Female | 1.523 | 1.094-2.120 |  |  | 1.412 | 0.993-2.008 |  |
| **Tumor size** |  |  |  |  |  |  |  |
| ≤ 5 cm | 1.000 |  | 1.038 |  | 1.000 |  | 0.554 |
| > 5 cm | 1.038 | 0.713-1.512 |  |  | 1.126 | 0.761-1.664 |  |
| **Differentiation** |  |  |  |  |  |  |  |
| Poor | 1.000 |  | < 0.001 |  | 1.000 |  | < 0.001 |
| Moderate/high | 0.353 | 0.249-0.501 |  |  | 0.372 | 0.255-0.542 |  |
| **Lymph node metastasis** | | |  |  |  |  |  |
| Negative | 1.000 |  | < 0.001 |  | 1.000 |  | < 0.001 |
| Positive | 2.931 | 2.069-4.153 |  |  | 2.707 | 1.876-3.905 |  |
| **Distant metastasis** | |  |  |  |  |  |  |
| M0 | 1.000 |  | 0.048 |  | 1.000 |  | 0.021 |
| M1 | 2.060 | 1.008-4.214 |  |  | 2.336 | 1.139-4.792 |  |
| **TNM stage** |  |  |  |  |  |  |  |
| I/II | 1.000 |  | < 0.001 |  | 1.000 |  | < 0.001 |
| III/IV | 2.935 | 2.077-4.147 |  |  | 2.746 | 1.911-3.945 |  |

^*^*P* values are from Log-rank test. ^†^CI: confidence interval.
