# Supplementary material for: TRIM21-mediated PRMT1 degradation attenuates colorectal cancer malignant progression
Source: Cell Death Dis. 2025 Jan 31;16(1):56. doi: 10.1038/s41419-025-07383-9 (PMC11785787; doi:10.1038/s41419-025-07383-9)
Supplement: Supplementary file 2 — Supplymentary Table2 [file 41419_2025_7383_MOESM2_ESM.docx]

**Table S2** Multivariate Cox regression analysis of overall survival and disease-specific survival of CRC patients.

| **Variables** | **Overall survival** | | |  | **Disease-specific survival** | | |
| --- | --- | --- | --- | --- | --- | --- | --- |
|  | **Hazard ratio** | **95% CI**^†^ | ***P^*^*** |  | **Hazard ratio** | **95% CI**^†^ | ***P^*^*** |
| **TRIM21** |  |  |  |  |  |  |  |
| Low | 1.000 |  | <0.001 |  | 1.000 |  | <0.001 |
| High | 0.341 | 0.220-0.528 |  |  | 0.350 | 0.222-0.552 |  |
| **Gender** |  |  |  |  |  |  |  |
| Male | 1.000 |  | <0.001 |  | 1.000 |  | 0.008 |
| Female | 1.817 | 1.275-2.591 |  |  | 1.665 | 1.142-2.427 |  |
| **Tumor size** |  |  |  |  |  |  |  |
| ≤ 5 cm | 1.000 |  | 0.184 |  | 1.000 |  | 0.390 |
| > 5 cm | 0.761 | 0.508-1.139 |  |  | 0.831 | 0.545-1.267 |  |
| **Differentiation** | |  |  |  |  |  |  |
| Poor | 1.000 |  | <0.001 |  | 1.000 |  | <0.001 |
| Moderate/high | 0.442 | 0.304-0.642 |  |  | 0.472 | 0.315-0.708 |  |
| **Lymph node metastasis** | | |  |  |  |  |  |
| Negative | 1.000 |  | 0.162 |  | 1.000 |  | 0.140 |
| Positive | 7.416 | 0.446-123.324 |  |  | 8.355 | 0.499-140.037 |  |
| **Distant metastasis** | | |  |  |  |  |  |
| M0 | 1.000 |  | 0.264 |  | 1.000 |  | 0.194 |
| M1 | 3.144 | 0.421-23.493 |  |  | 3.812 | 0.505-28.767 |  |
| **TNM stage** |  |  |  |  |  |  |  |
| I/II | 1.000 |  | 0.438 |  | 1.000 |  | 0.361 |
| III/IV | 0.327 | 0.019-0.524 |  |  | 0.267 | 0.016-4.547 |  |

*^*^P* values are from Log-rank test; ^†^CI: confidence interval.
