# Supplementary figures and images for: TRIM21-mediated PRMT1 degradation attenuates colorectal cancer malignant progression
Source: Cell Death Dis. 2025 Jan 31;16(1):56. doi: 10.1038/s41419-025-07383-9 (PMC11785787; doi:10.1038/s41419-025-07383-9)

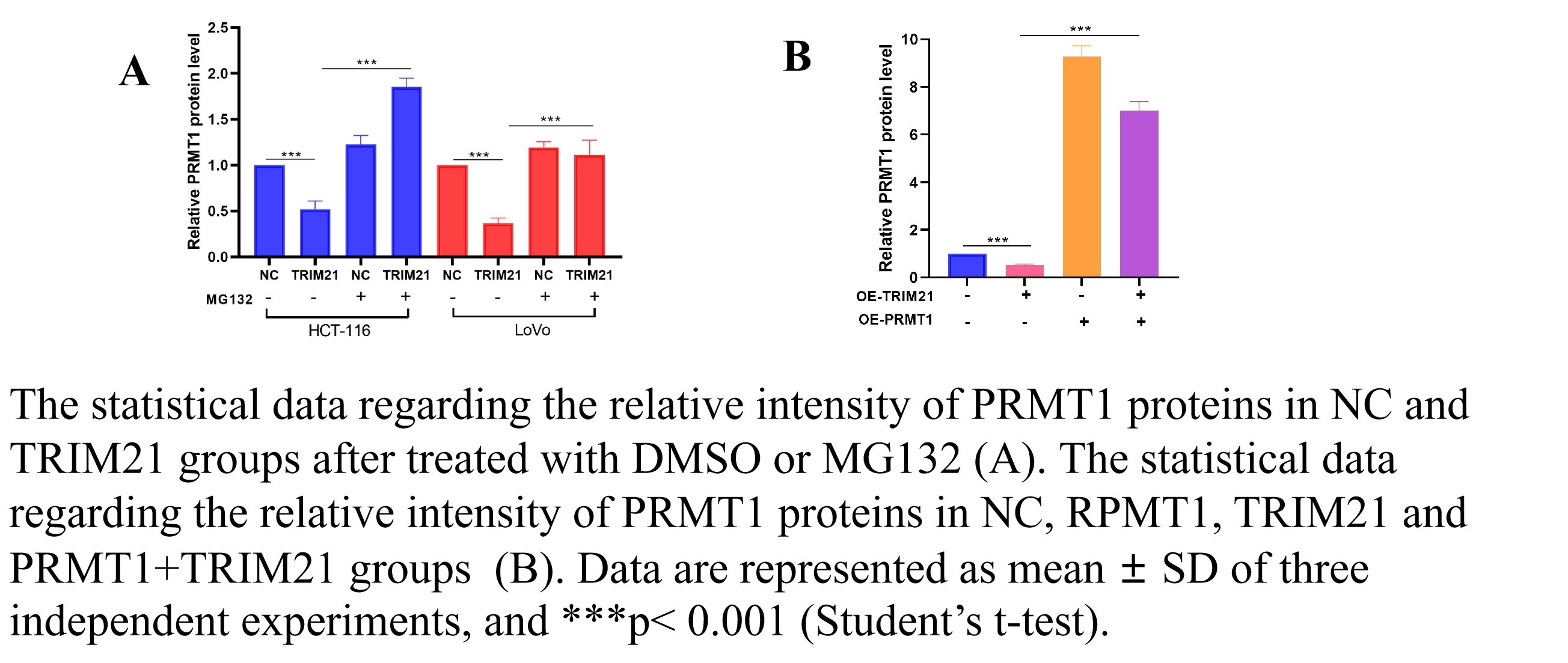

Supplement: Supplementary file 3 — Supplymentary Figure 1 [file 41419_2025_7383_MOESM3_ESM.jpg]
