# Supplementary material for: TRIM21-mediated PRMT1 degradation attenuates colorectal cancer malignant progression
Source: Cell Death Dis. 2025 Jan 31;16(1):56. doi: 10.1038/s41419-025-07383-9 (PMC11785787; doi:10.1038/s41419-025-07383-9)
Supplement: Supplementary file 4 — Supplementary Material and Methods [file 41419_2025_7383_MOESM4_ESM.doc]

**Supplementary Materials for**

**TRIM21-mediated PRMT1 degradation attenuates colorectal cancer malignant progression**

**Supplementary Figure legends**

**Figure S1.** The statistical data regarding the relative intensity of PRMT1 proteins in NC and TRIM21 groups after treated with DMSO or MG132 (A). The statistical data regarding the relative intensity of PRMT1 proteins in NC, RPMT1, TRIM21 and PRMT1+TRIM21 groups (B). Data are represented as mean ± SD of three independent experiments, and ***p< 0.001 (Student’s t-test).
