# Supplementary material for: TRIM21-mediated PRMT1 degradation attenuates colorectal cancer malignant progression
Source: Cell Death Dis. 2025 Jan 31;16(1):56. doi: 10.1038/s41419-025-07383-9 (PMC11785787; doi:10.1038/s41419-025-07383-9)

Figure 2E HCT-116

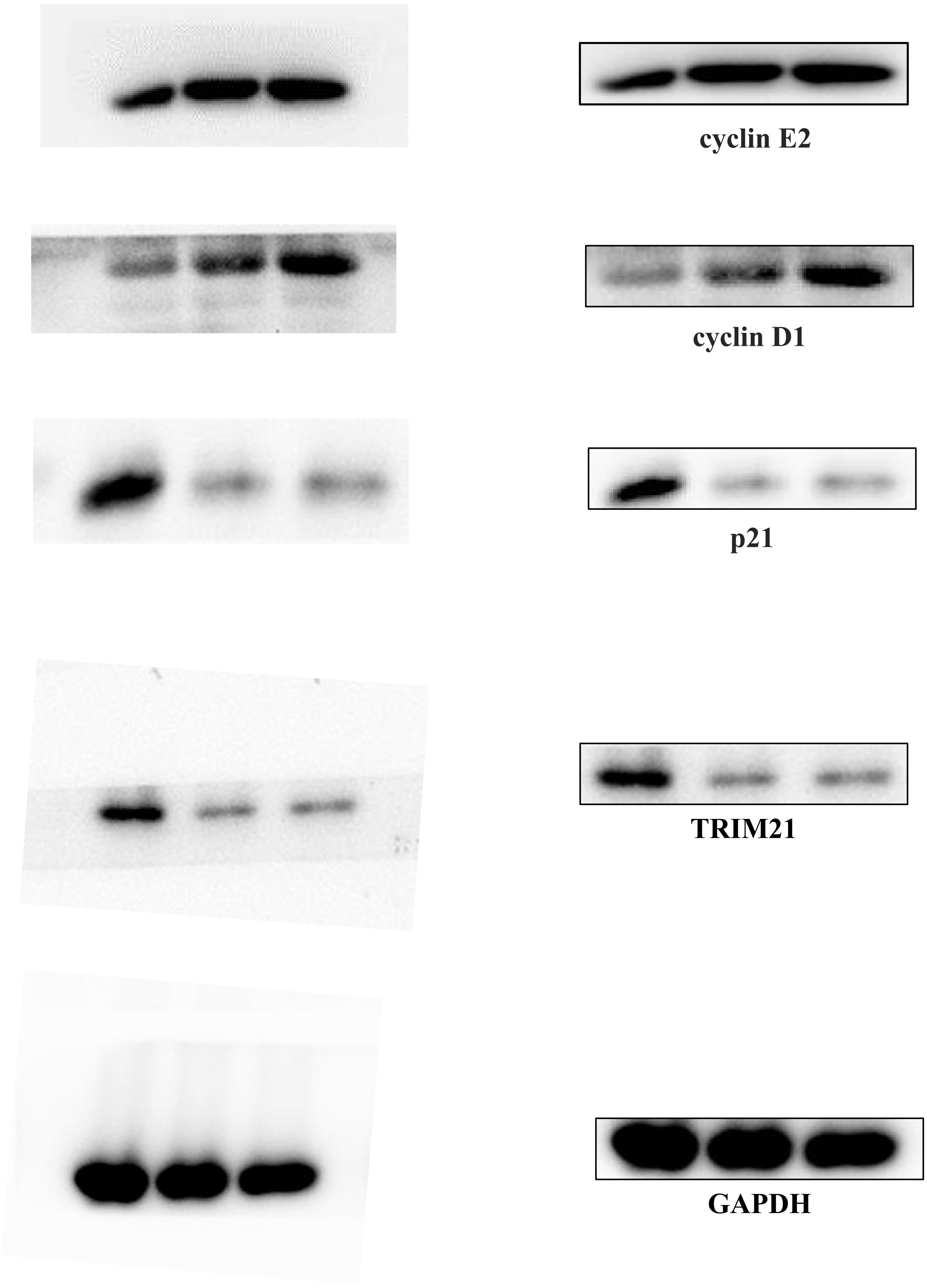

Figure 2E LoVo

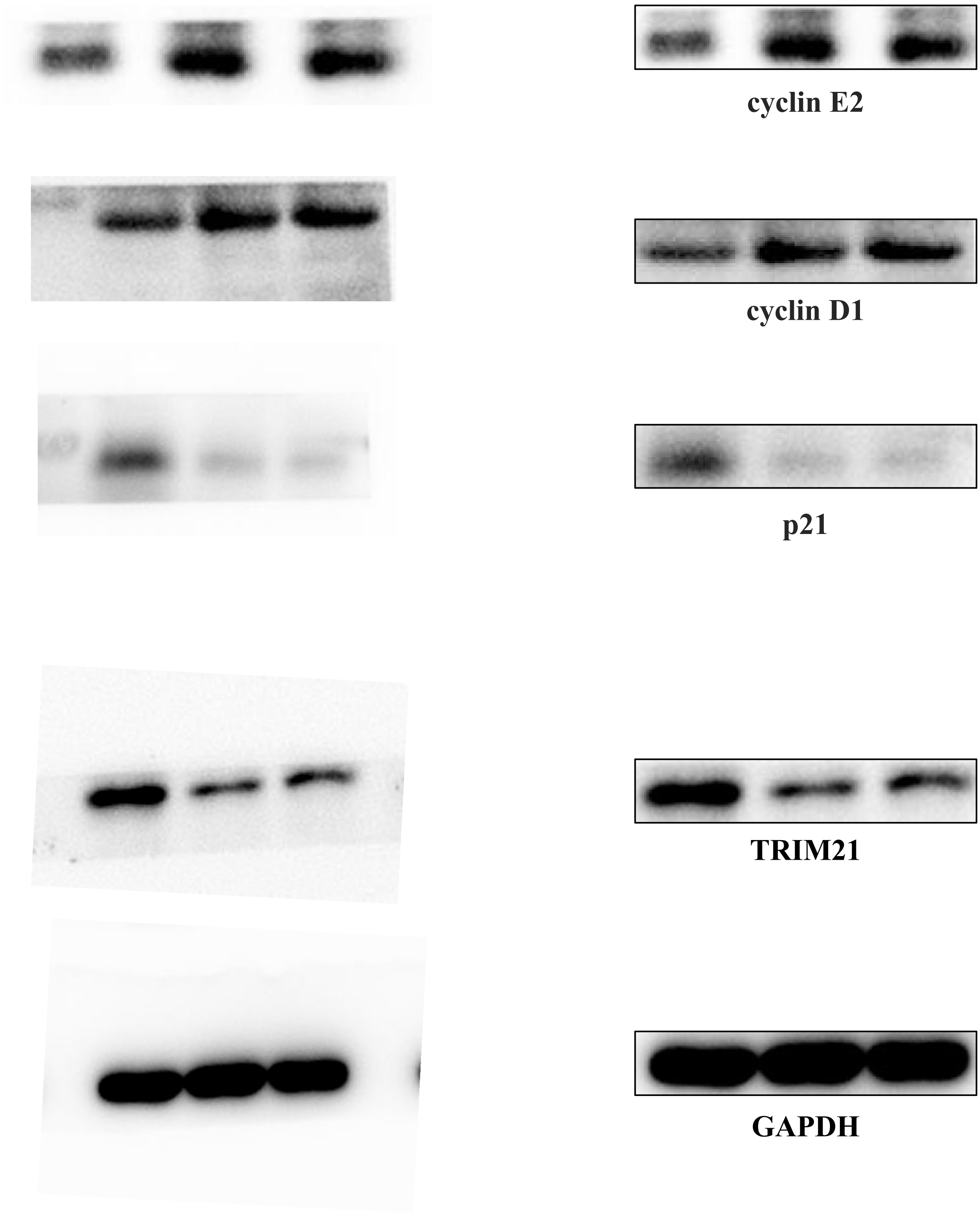

Figure 2F HCT-116

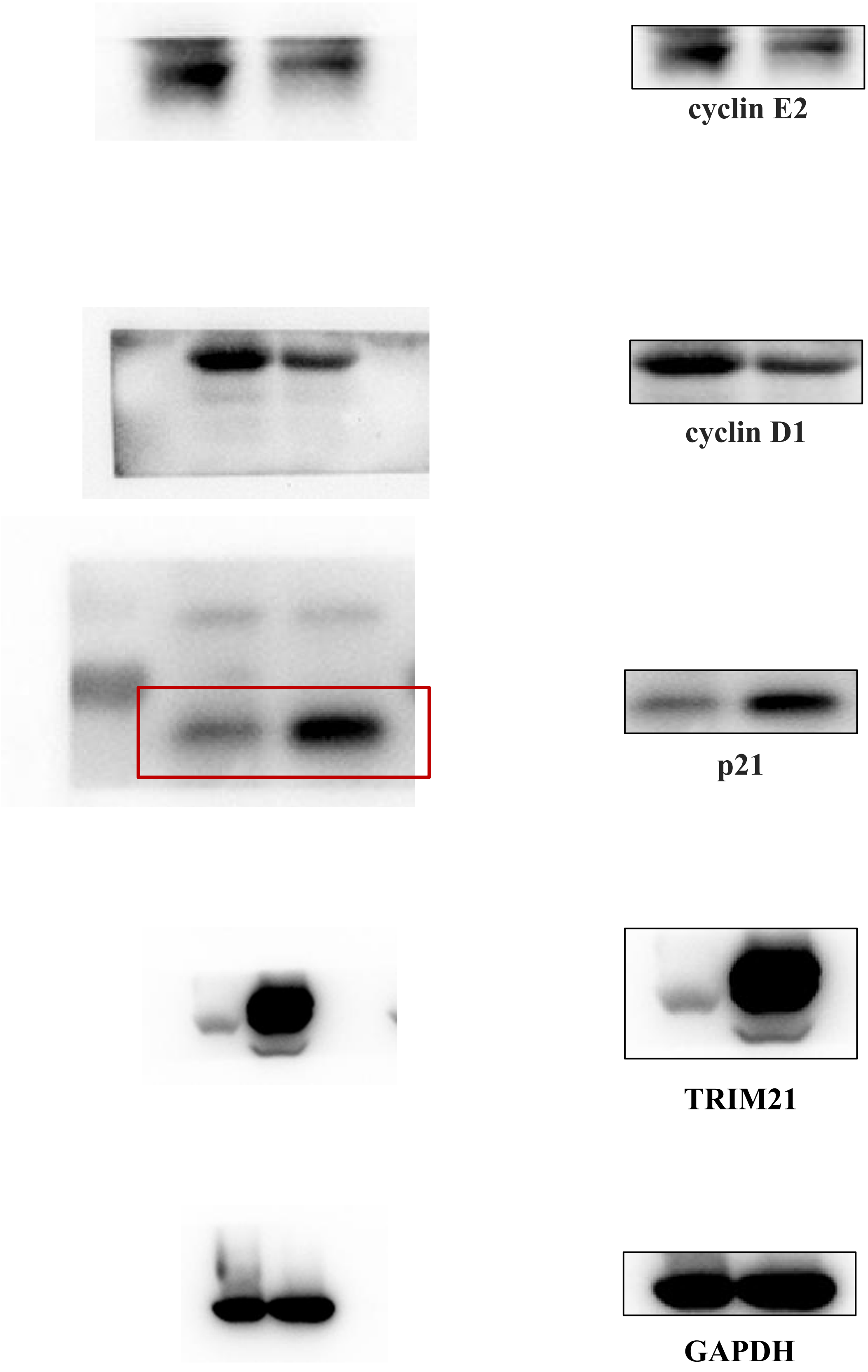

Figure 2F LoVo

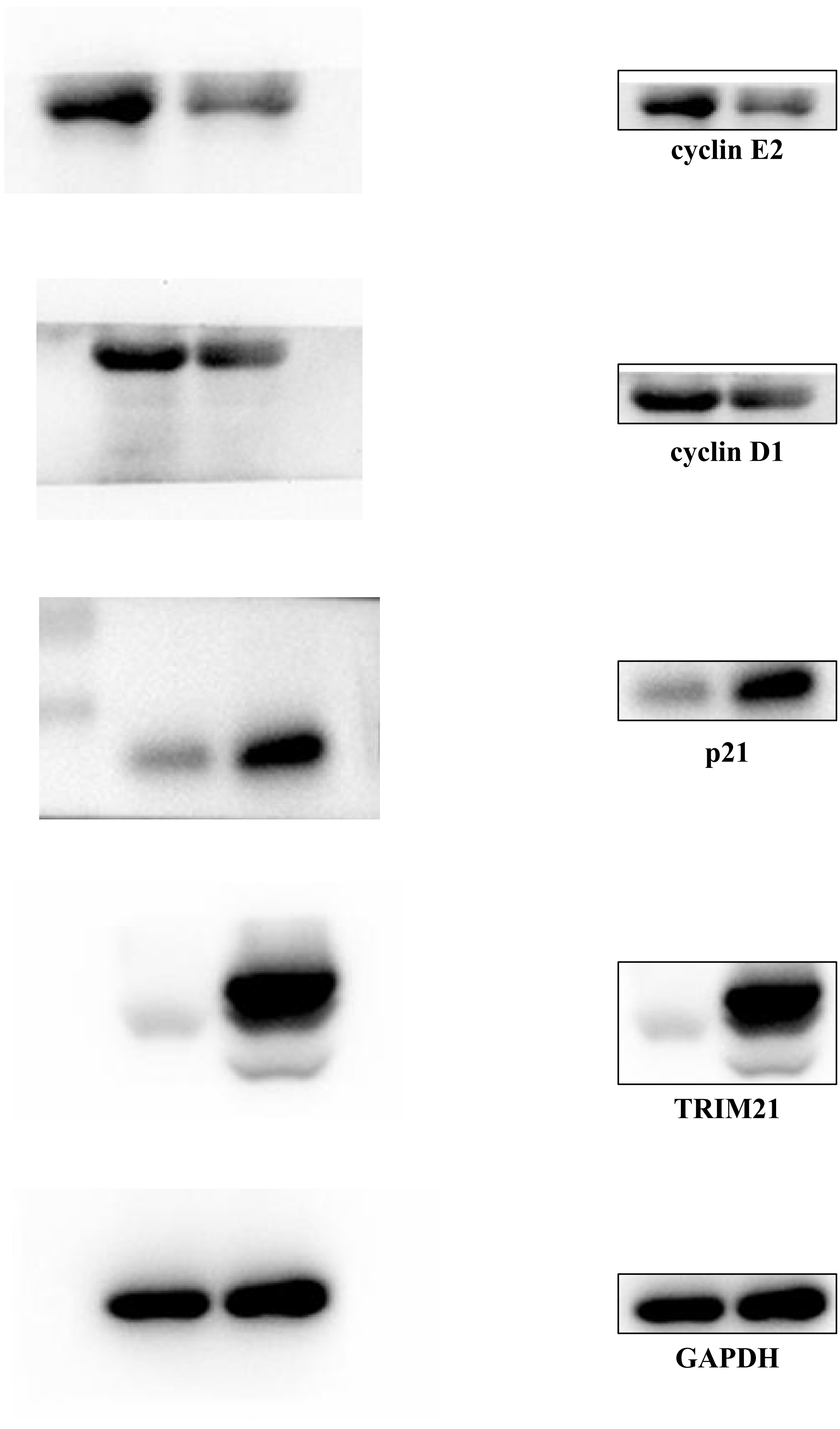

Figure 3E HCT-116

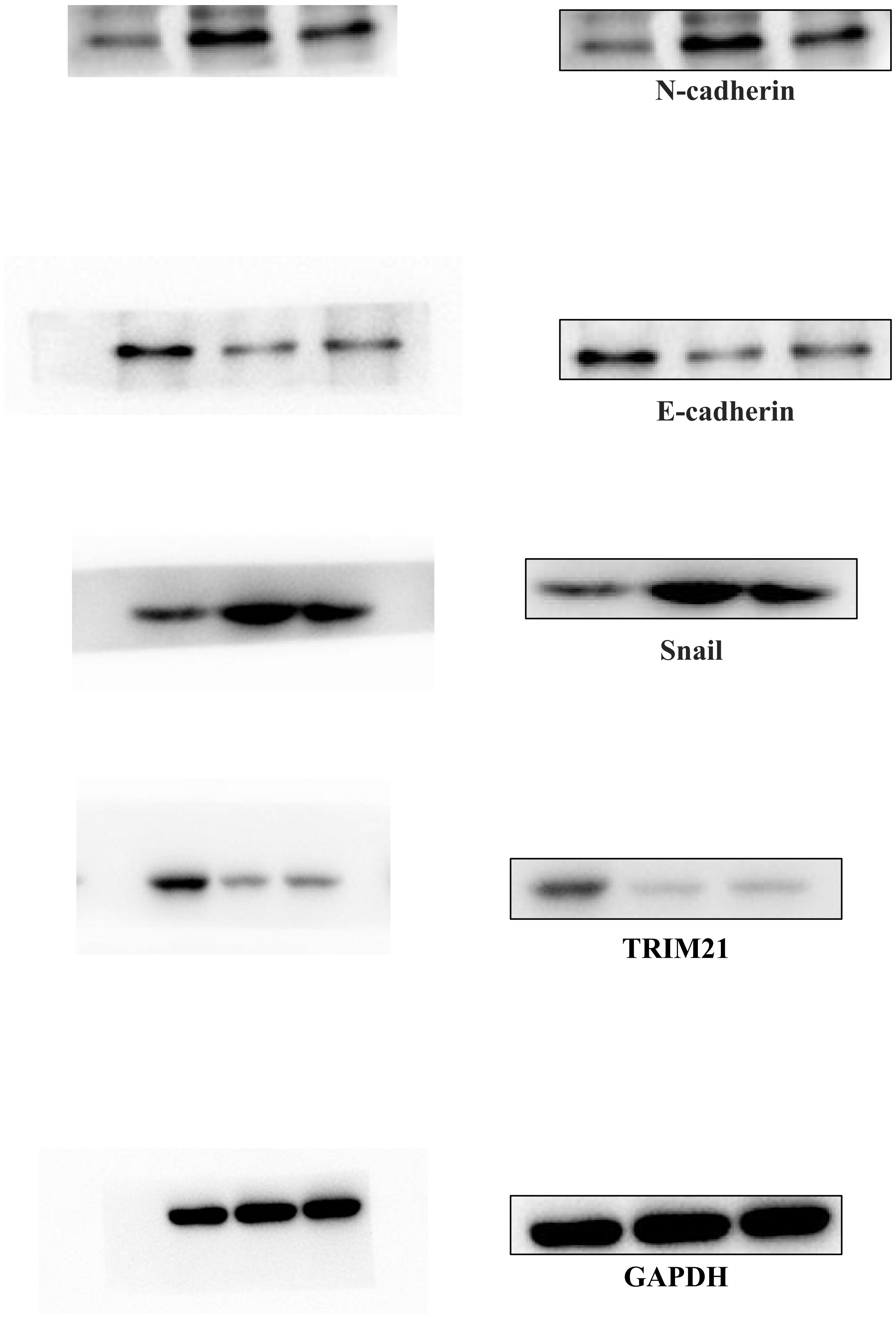

Figure 3E LoVo

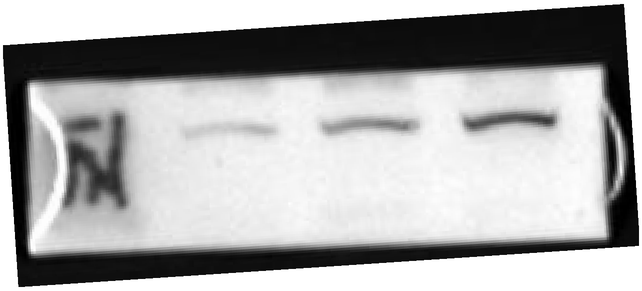

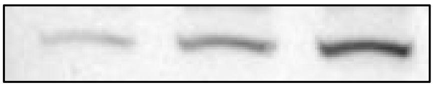

N-cadherin

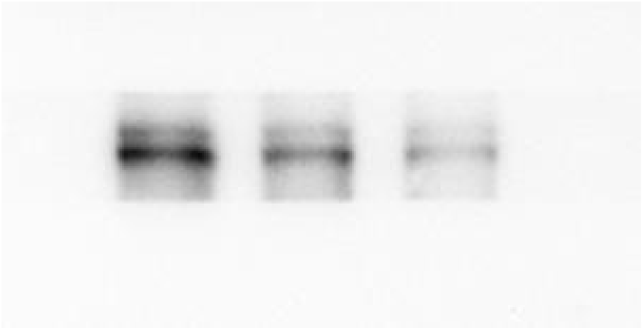

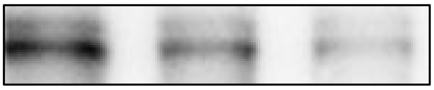

E-cadherin

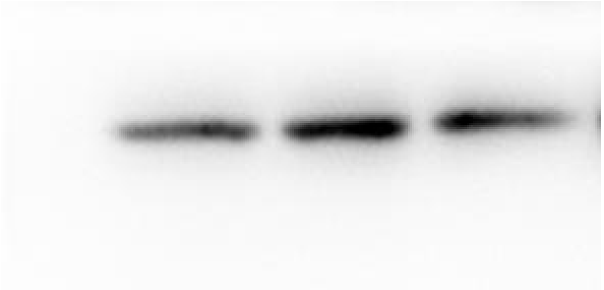

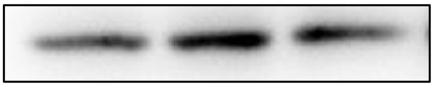

Snail

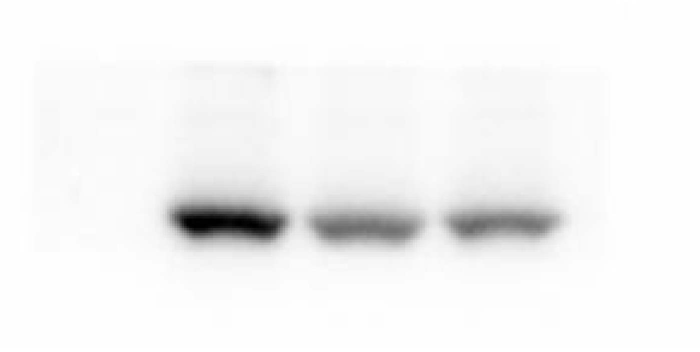

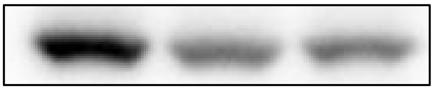

TRIM21

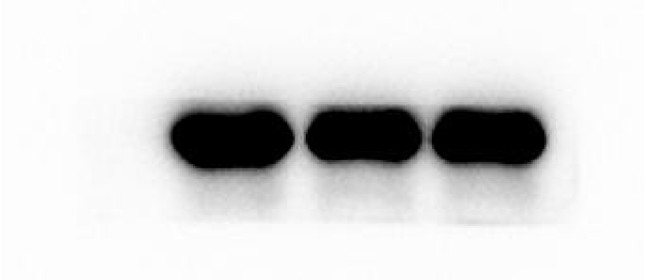

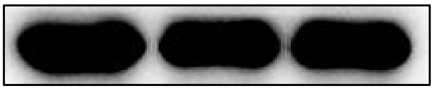

GAPDH

Figure 3F HCT-116

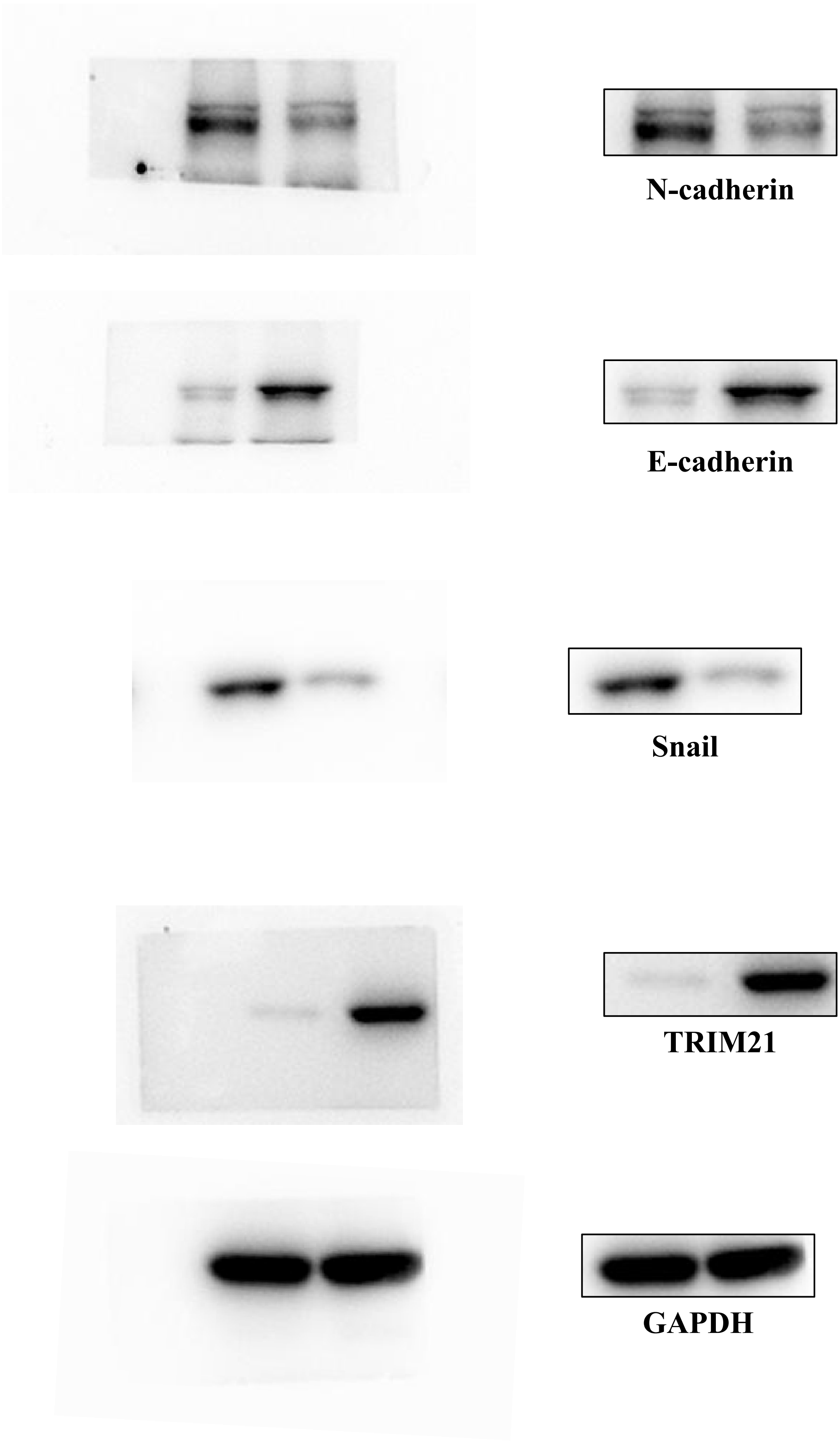

Figure 3F LoVo

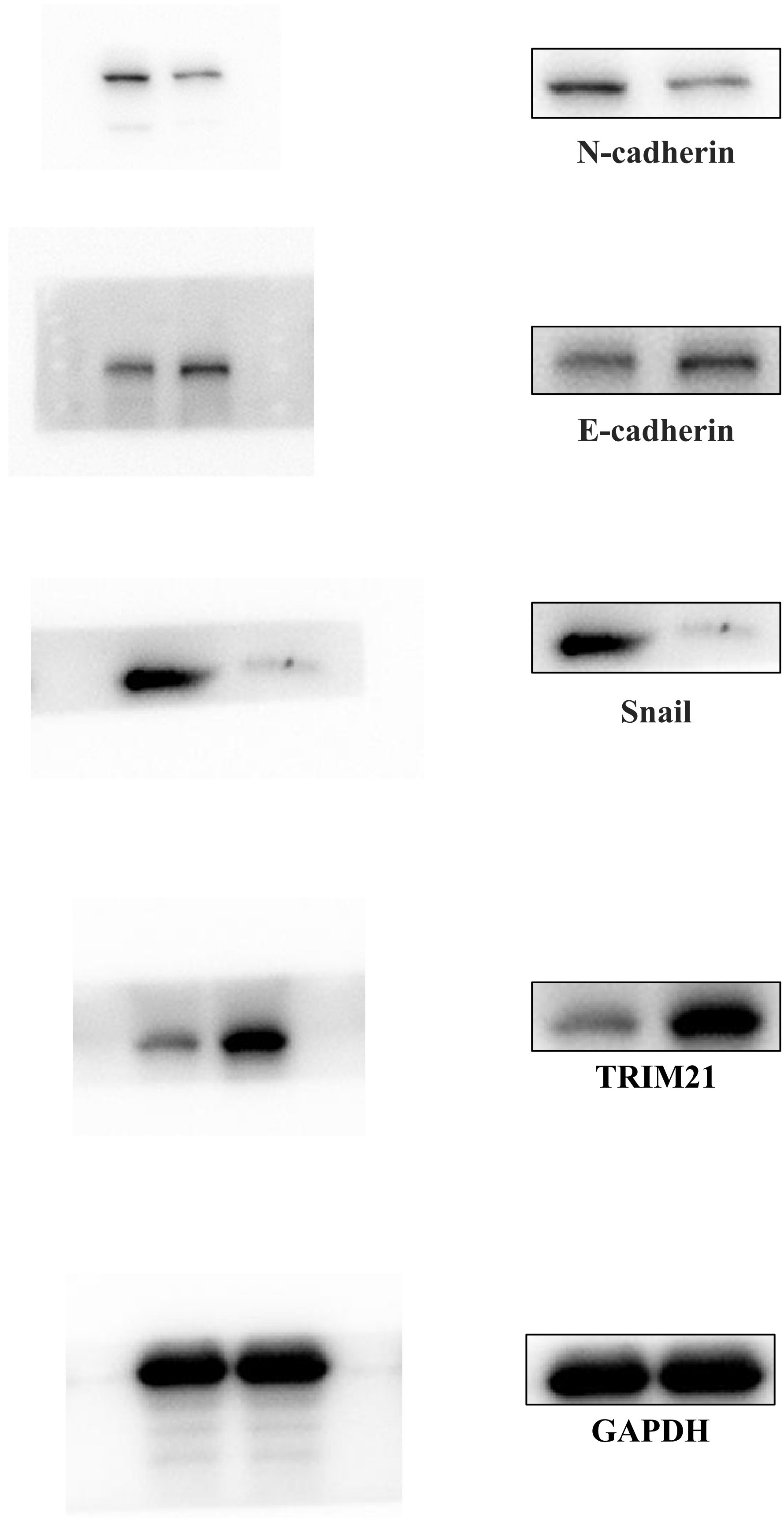

Figure 4A HCT-116

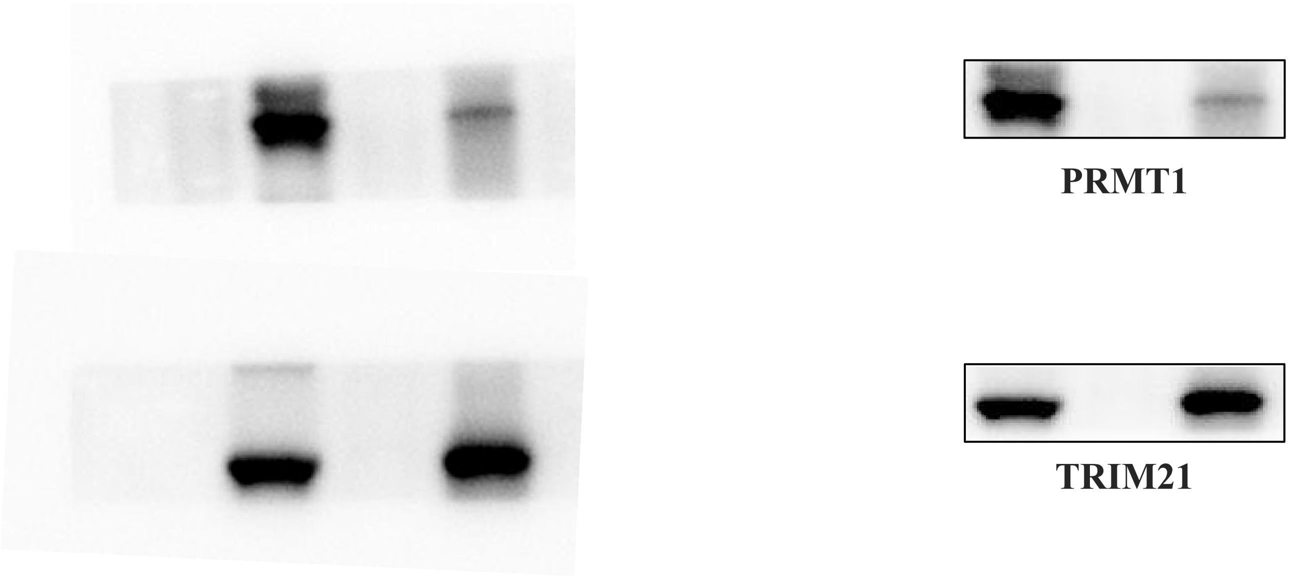

Figure 4A LoVo

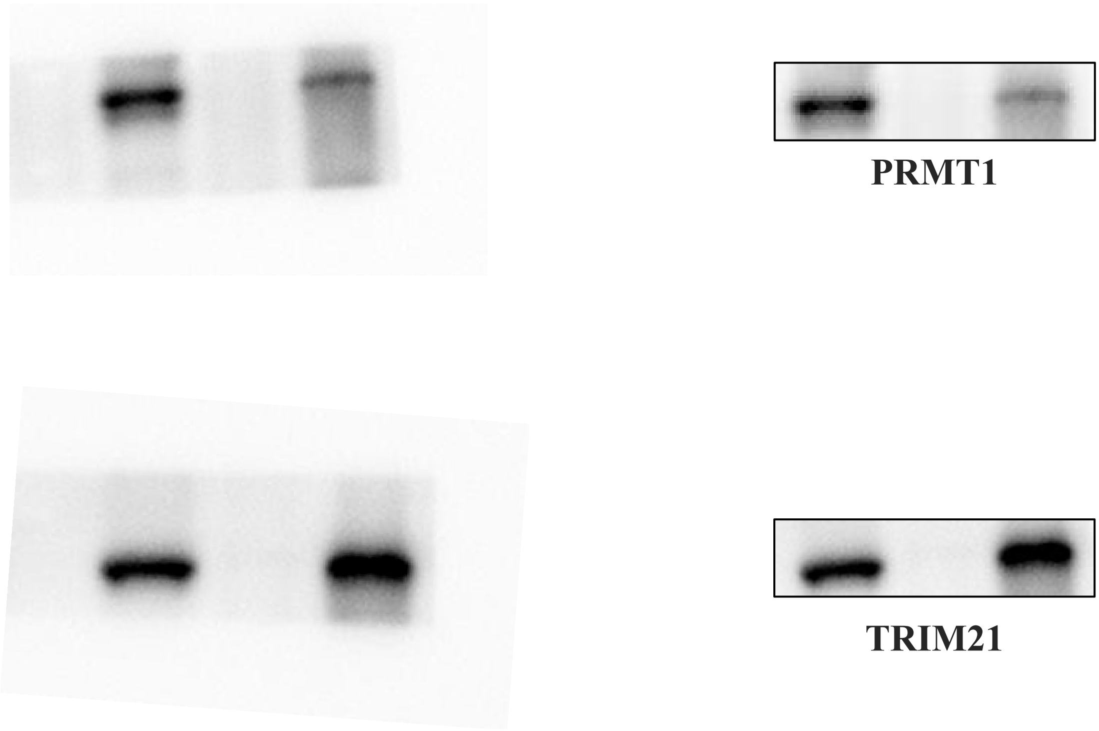

Figure 4B HCT-116

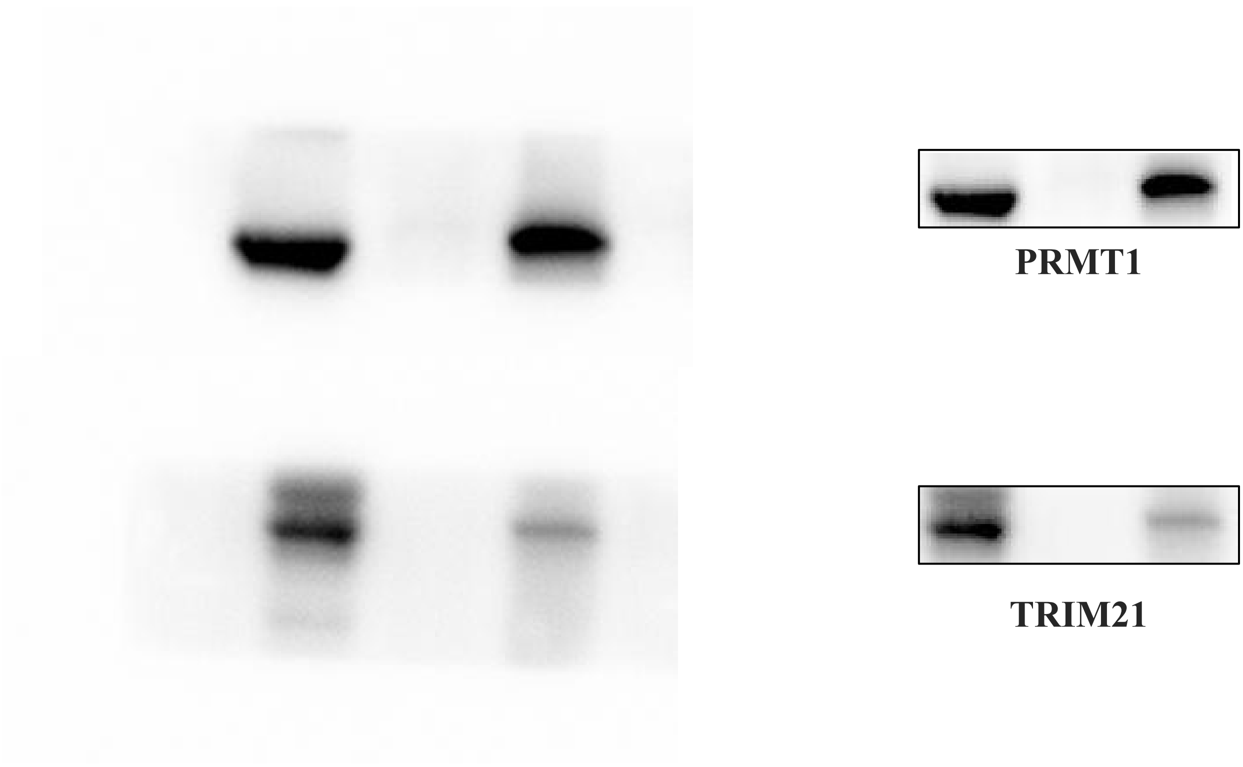

Figure 4B LoVo

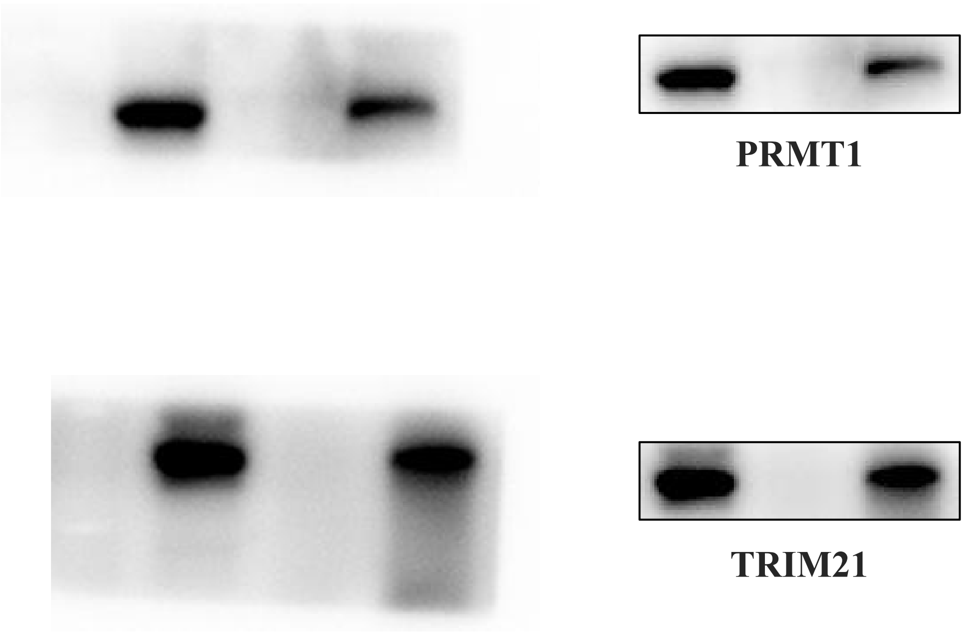

Figure 4C HCT-116

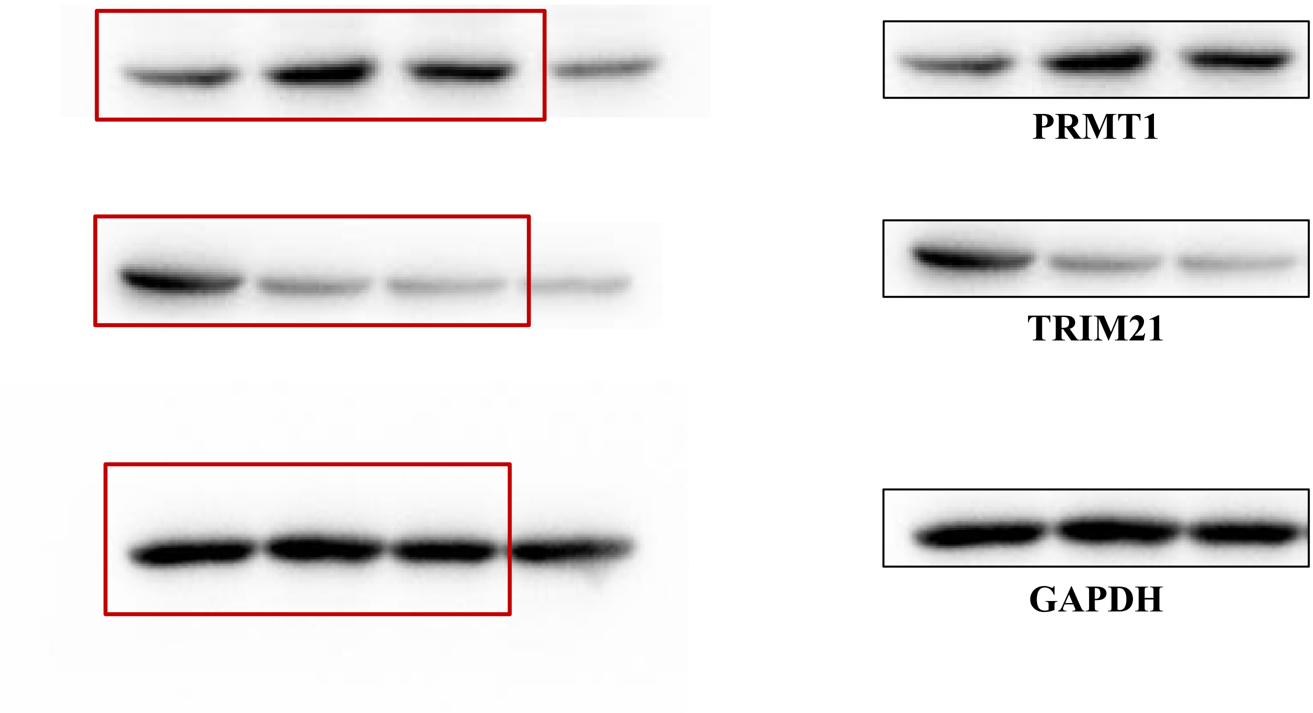

Figure 4C LOVO

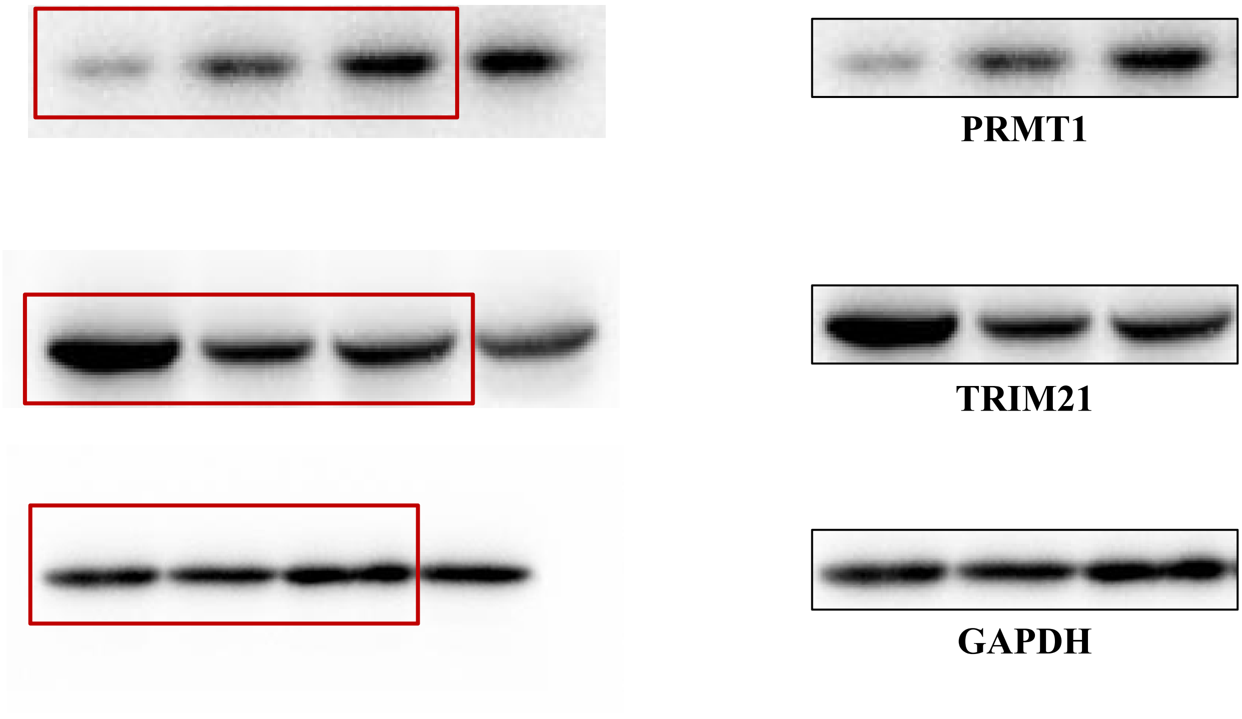

Figure 4D-HCT116

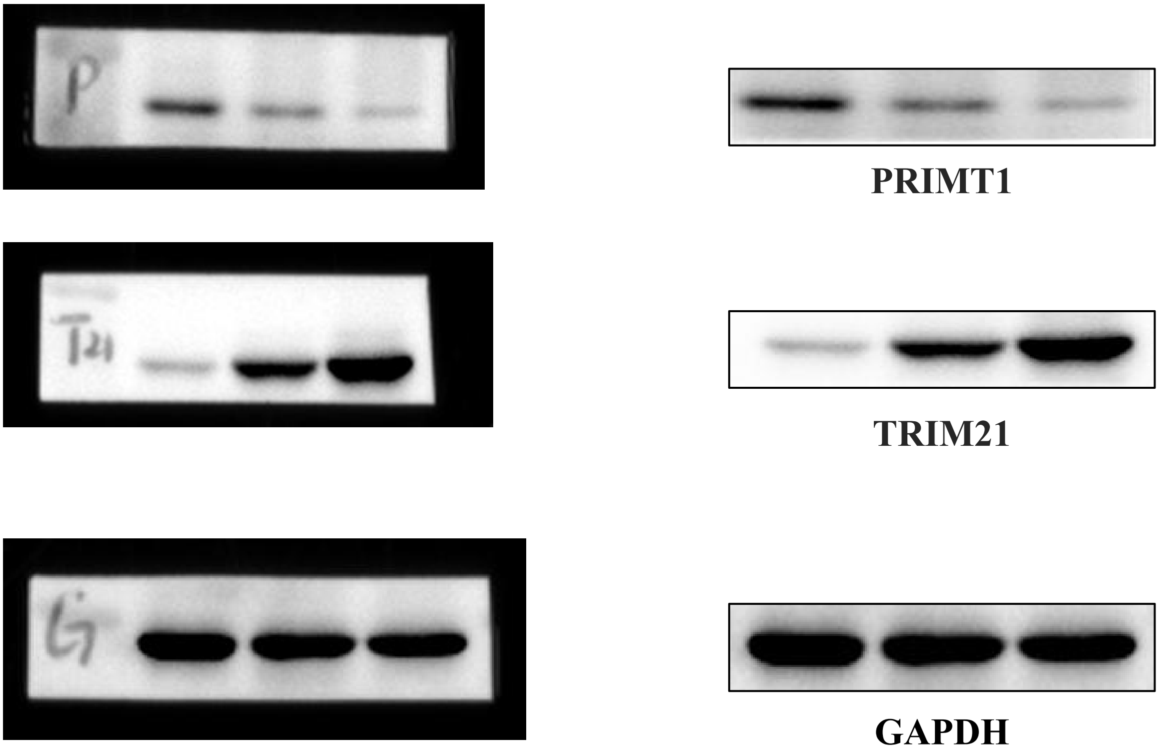

Figure 4D-LOVO

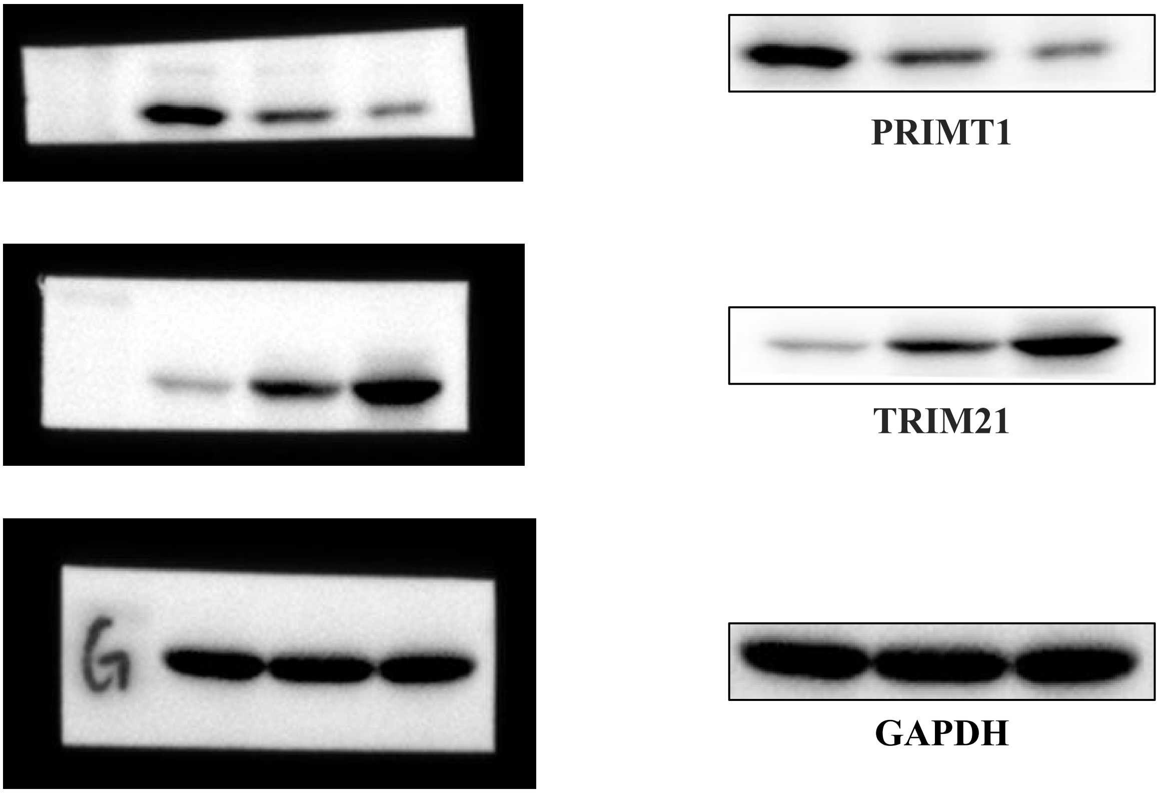

Figure 4G

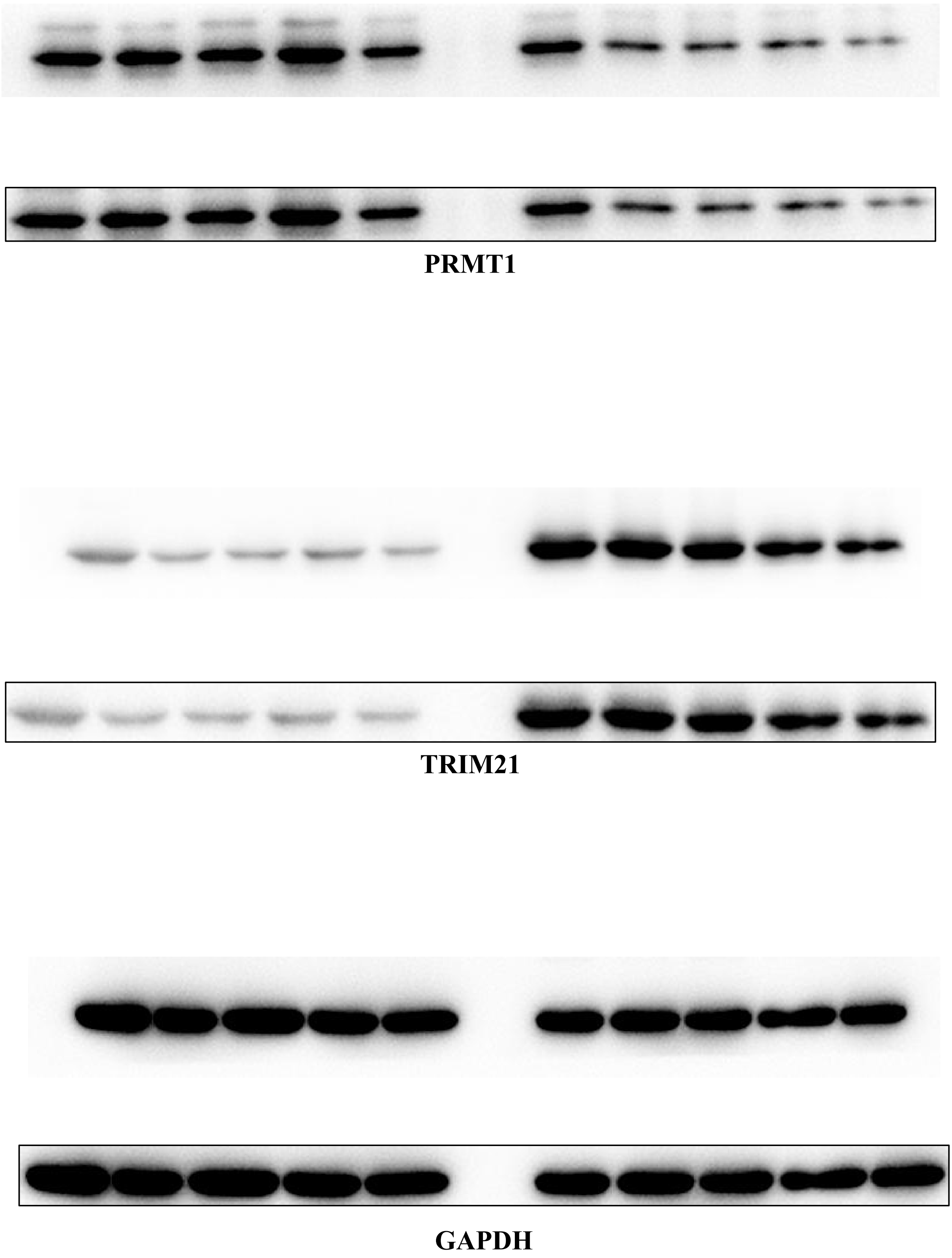

Figure 5A HCT-116

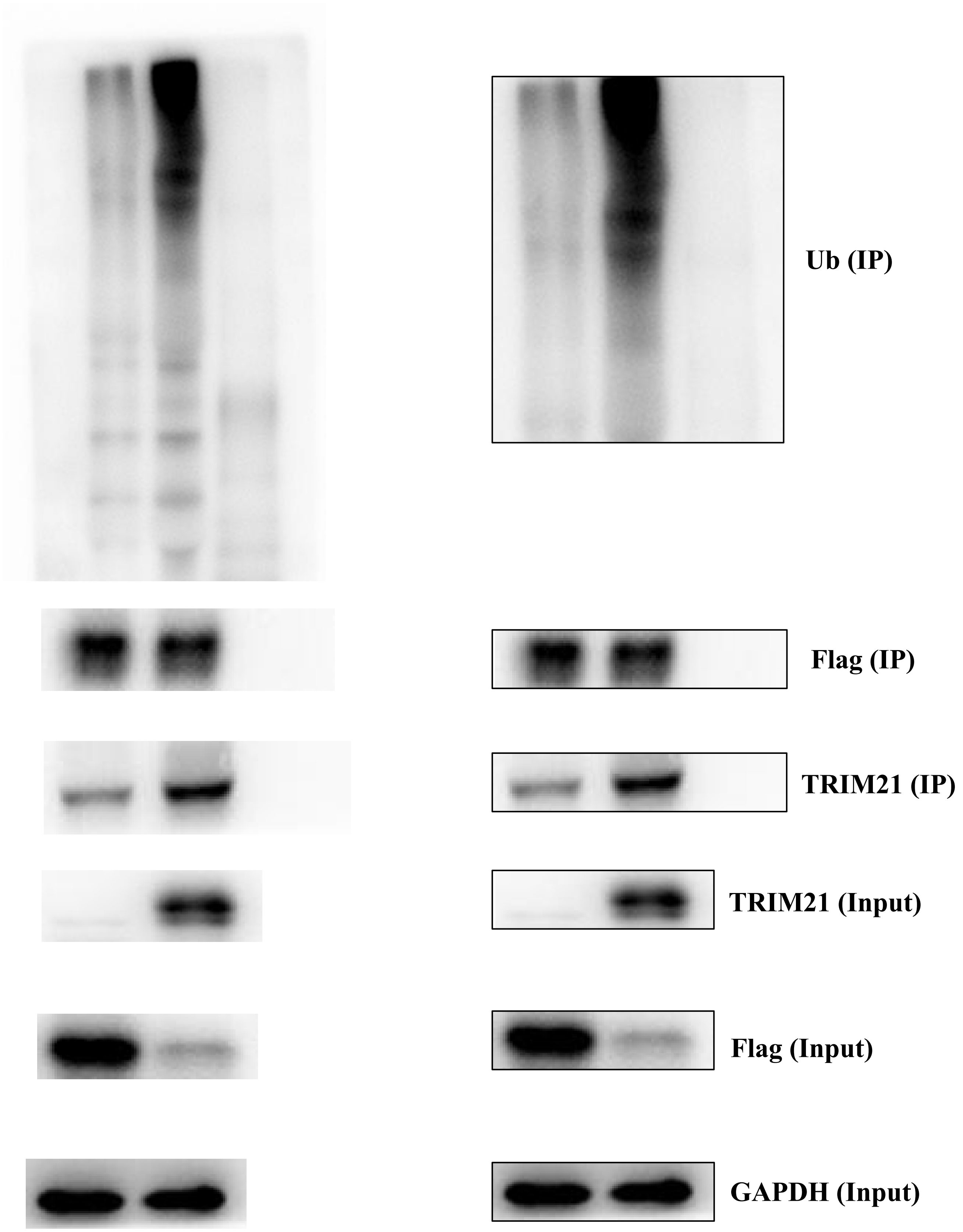

Figure 5A LoVo

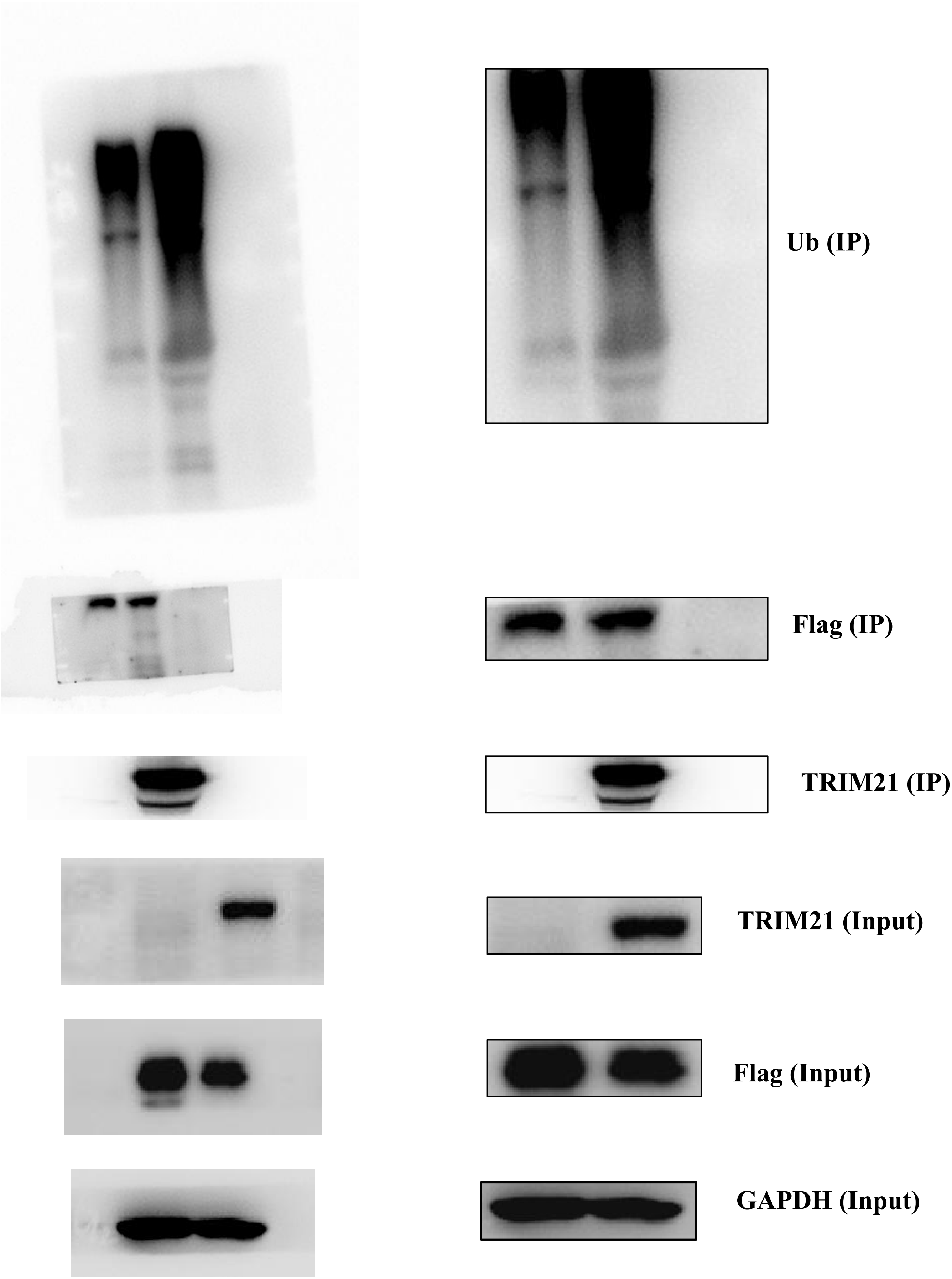

Figure 5B HCT-116

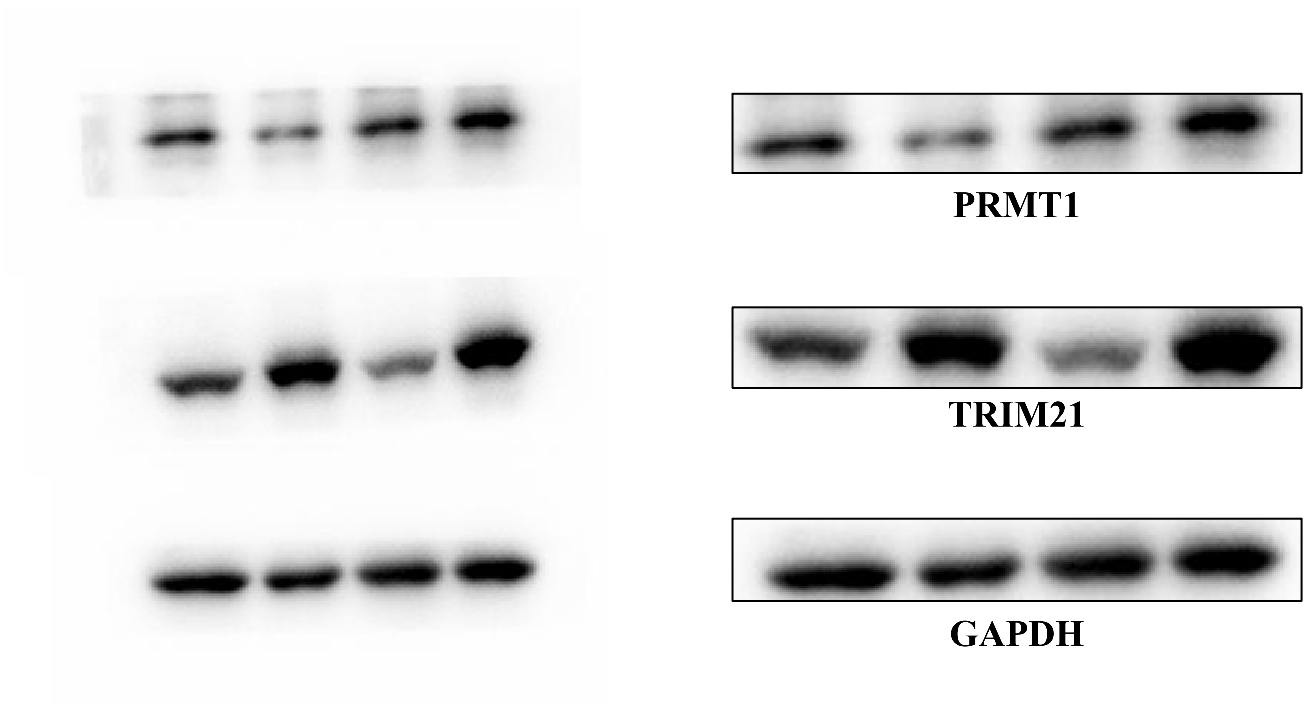

Figure 5B LoVo

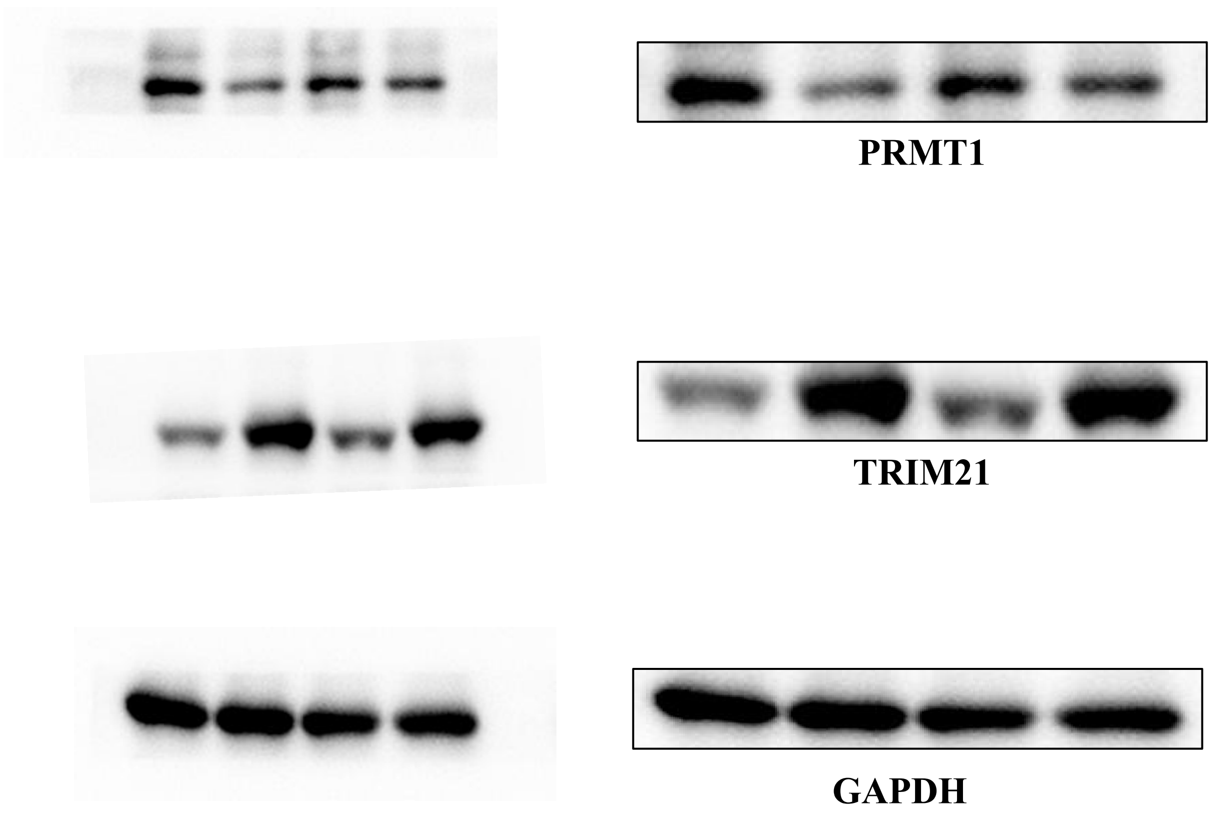

Figure 5D

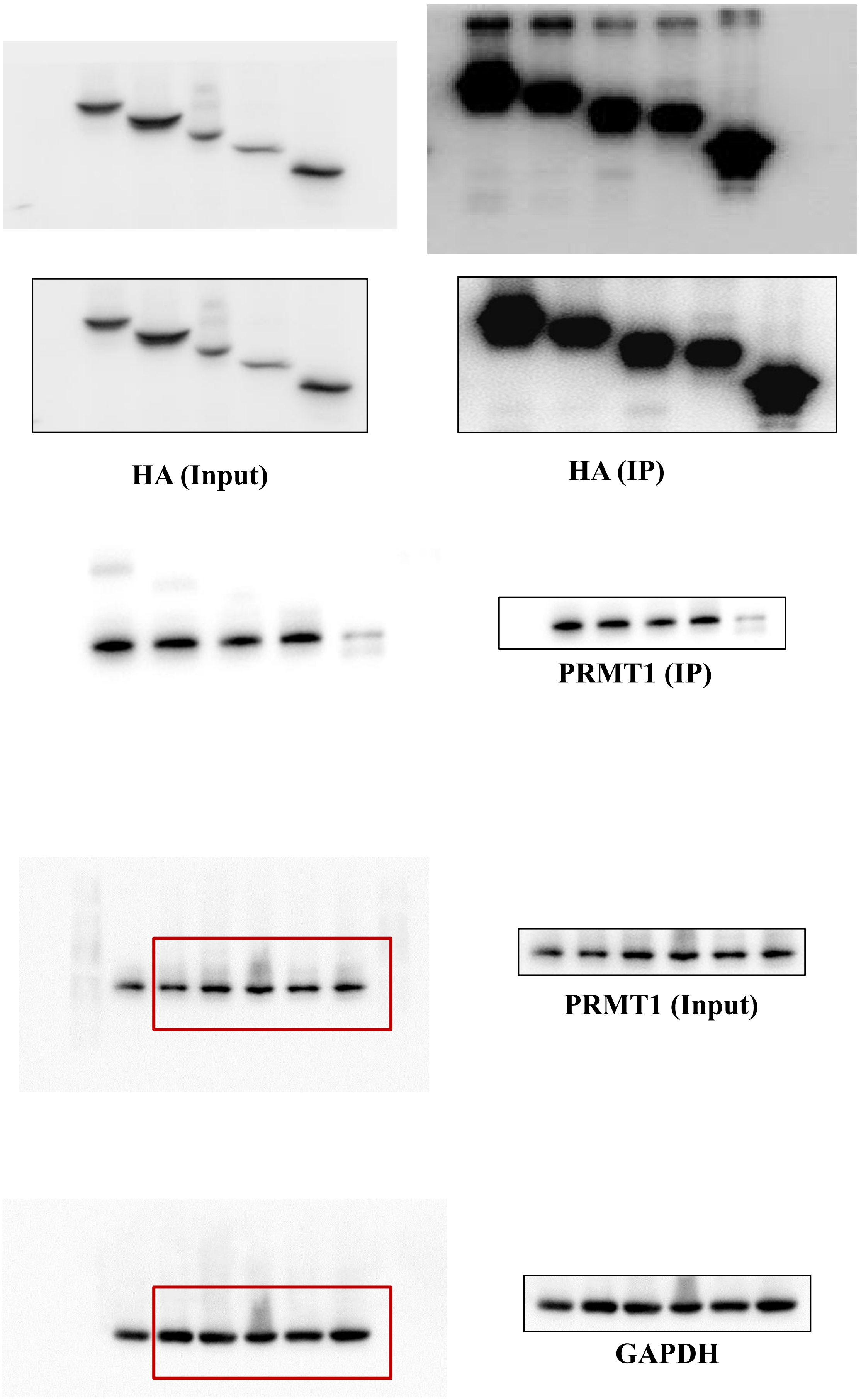

Figure 5E-Input

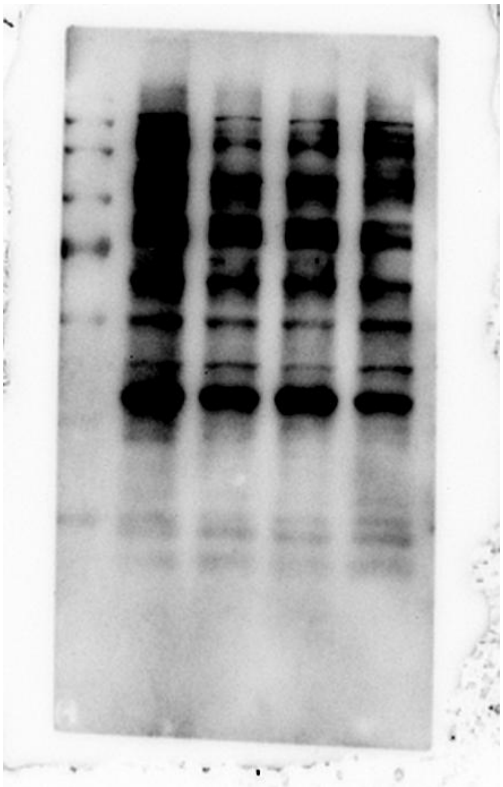

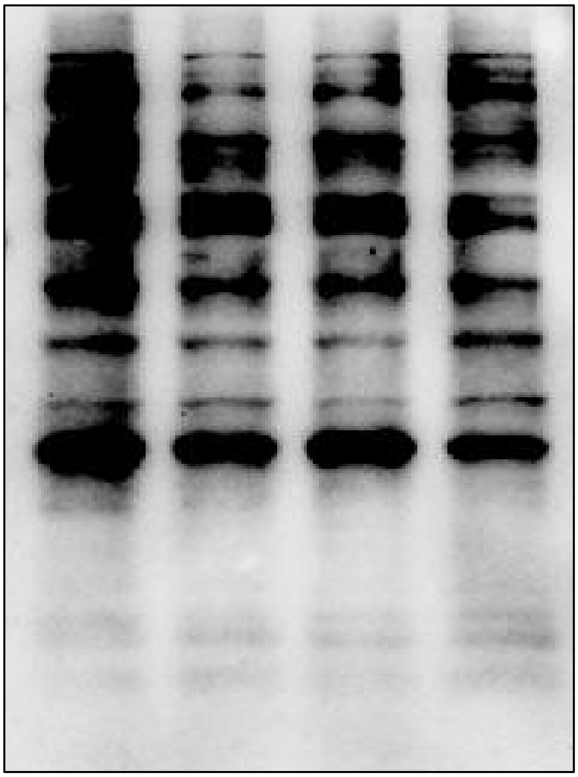

His

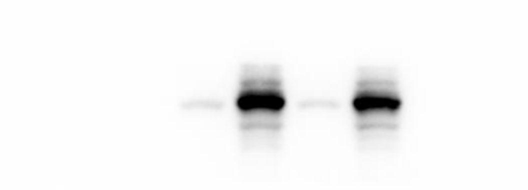

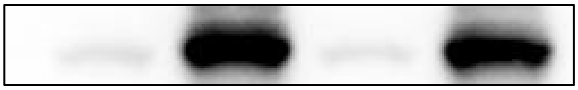

TRIM21

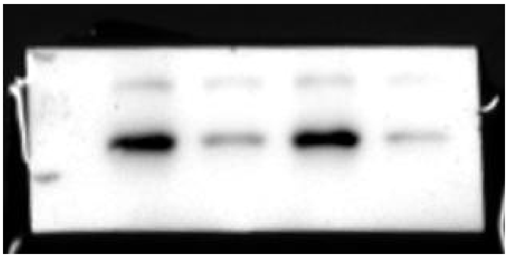

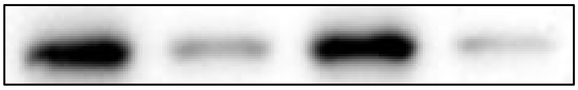

myc

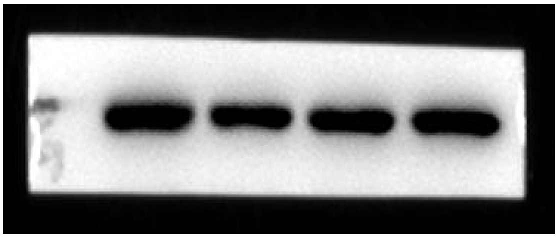

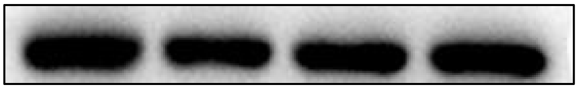

GAPDH

Figure 5E-IP

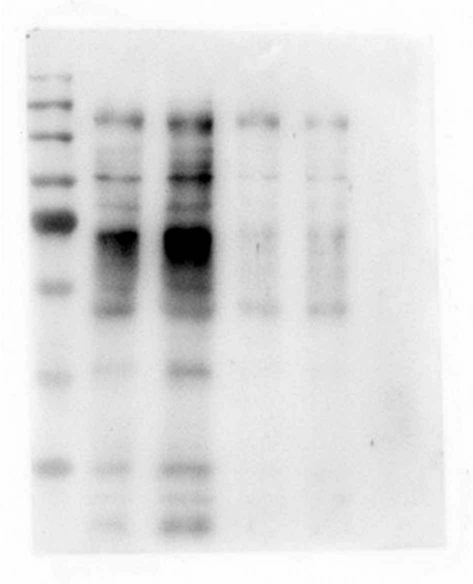

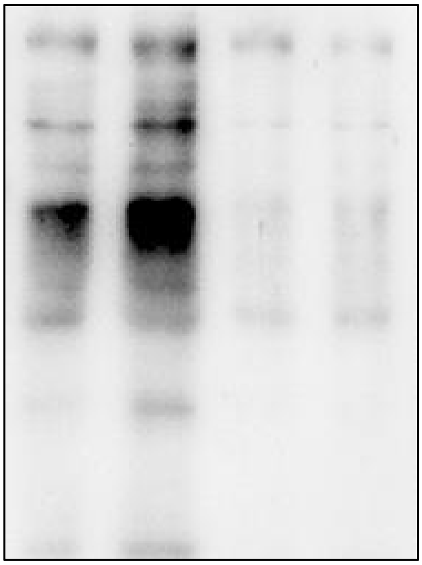

His

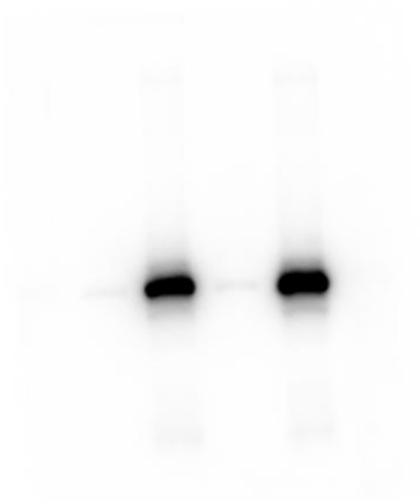

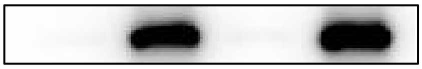

TRIM21

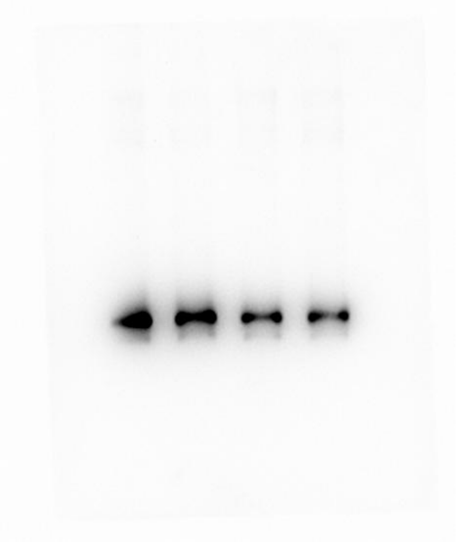

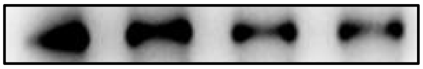

myc

Figure 6A

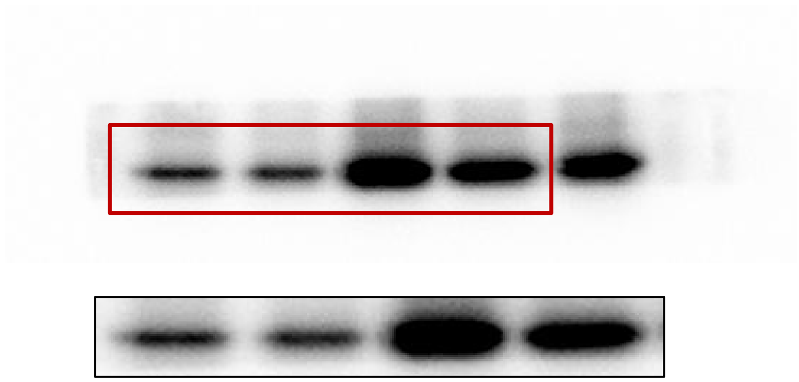

PRMT1

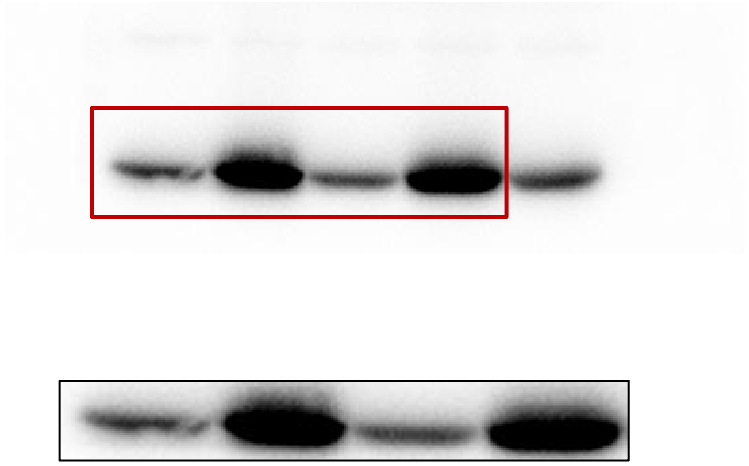

TRIM21

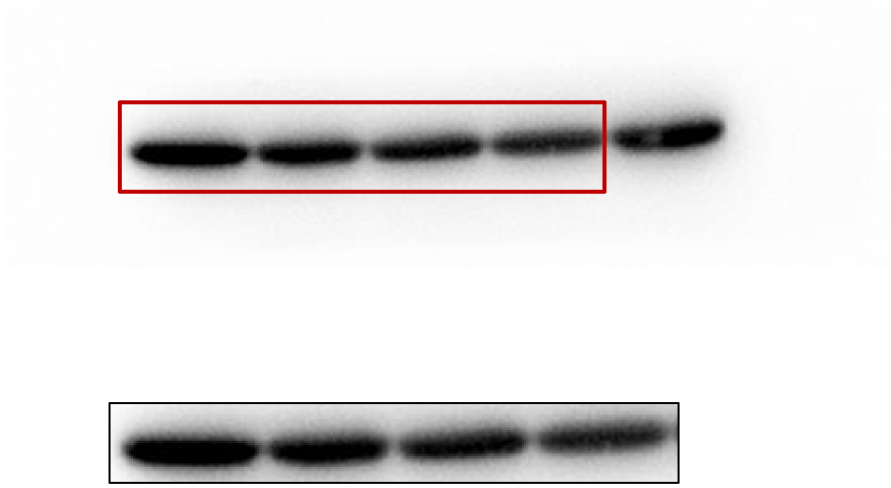

GAPDH

Figure 6D

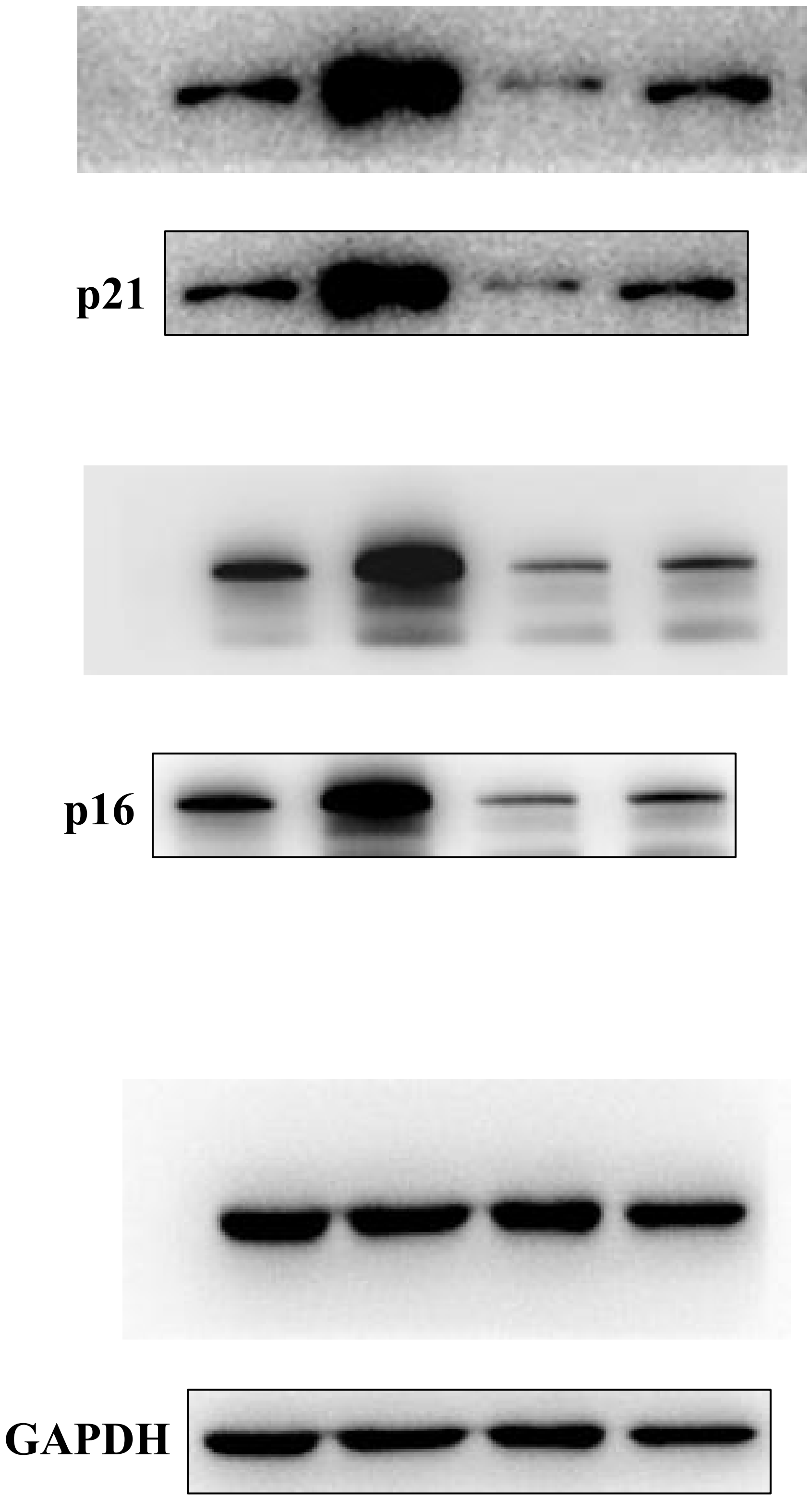

Supplement: Supplementary file 5 — uncropped WB Raw Data [file 41419_2025_7383_MOESM5_ESM.pdf]
